# Phosphatidylserine synthase in the endoplasmic reticulum of *Toxoplasma* is essential for its lytic cycle in human cells

**DOI:** 10.1016/j.jlr.2024.100535

**Published:** 2024-03-24

**Authors:** Dimitrios Alexandros Katelas, Rosalba Cruz-Miron, Ruben D. Arroyo-Olarte, Jos F. Brouwers, Ratnesh Kumar Srivastav, Nishith Gupta

**Affiliations:** 1 Department of Molecular Parasitology, Faculty of Life Sciences, Humboldt University, Berlin, Germany; 2 Intracellular Parasite Education and Research Labs (iPEARL), Department of Biological Sciences, Birla Institute of Technology and Science, Pilani (BITS-Pilani), Hyderabad, India; 3 Carrera de Médico Cirujano y Unidad de Biomedicina (UBIMED), FES-Iztacala, Universidad Nacional Autónoma de México, Tlalnepantla, Mexico; 4 Analysis Techniques in the Life Sciences, Centre of Expertise Perspective in Health, Avans University of Applied Sciences, Breda, The Netherlands

## Abstract

Glycerophospholipids have emerged as a significant contributor to the intracellular growth of pathogenic protist *Toxoplasma gondii*. Phosphatidylserine (PtdSer) is one such lipid, attributed to the locomotion and motility-dependent invasion and egress events in its acutely infectious tachyzoite stage. However, the de novo synthesis of PtdSer and the importance of the pathway in tachyzoites remain poorly understood. We show that a base-exchange-type PtdSer synthase (PSS) located in the parasite’s endoplasmic reticulum produces PtdSer, which is rapidly converted to phosphatidylethanolamine (PtdEtn) by PtdSer decarboxylase (PSD) activity. The PSS-PSD pathway enables the synthesis of several lipid species, including PtdSer (16:0/18:1) and PtdEtn (18:2/20:4, 18:1/18:2 and 18:2/22:5). The PSS-depleted strain exhibited a lower abundance of the major ester-linked PtdEtn species and concurrent accrual of host-derived ether-PtdEtn species. Most phosphatidylthreonine (PtdThr) species—an exclusive natural analog of PtdSer, also made in the endoplasmic reticulum—were repressed. PtdSer species, however, remained largely unaltered, likely due to the serine-exchange reaction of PtdThr synthase in favor of PtdSer upon PSS depletion. Not least, the loss of PSS abrogated the lytic cycle of tachyzoites, impairing the cell division, motility, and egress. 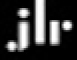 In a nutshell, our data demonstrate a critical role of PSS in the biogenesis of PtdSer and PtdEtn species and its physiologically essential repurposing for the asexual reproduction of a clinically relevant intracellular pathogen.

*Toxoplasma gondii*—a widespread intracellular pathogen of livestock, wildlife, and humans—belongs to the protozoan phylum Apicomplexa. Along with other apicomplexans, such as *Plasmodium, Eimeria*, and *Cryptosporidium* species, *T. gondii* is commonly used to decipher the molecular concepts of intracellular parasitism. The parasite infects a range of nucleated host cells, and its asexual reproduction is marked by recurring lytic cycles leading to tissue necrosis and acute infection (1). Lytic cycle of *T. gondii* starts with the active invasion of a host cell by extracellular parasites. Once intracellular, the parasite multiplies by endodyogeny, forming two progeny within a parent cell every 8–10 h. The progeny egress by lysing the dilapidated host cell and then invade adjacent cells, initiating a new lytic cycle. An acute infection due to tissue necrosis can be fatal in immune-deficient individuals. Among other factors, glycerophospholipids have emerged as the critical molecular determinants of *Toxoplasma*’s lytic cycle in the human host cells. Some phospholipids serve specialized functions during parasite infection beyond their conventional roles in membrane biogenesis (2–8), spurring a deep interest in the synthesis, salvage, sensing and trafficking of lipids in *T. gondii*.

The glycerophospholipid composition and biosynthesis in the tachyzoite stage of *T. gondii* are similar to human host cells, with a few notable exceptions (2, 9, 10). Briefly, phosphatidylcholine (PtdCho) is a dominant phospholipid in the parasite membrane, followed by phosphatidylethanolamine (PtdEtn). Other glycerophospholipids in the parasite include phosphatidylthreonine (PtdThr), phosphatidylinositol (PtdIns), phosphatidylserine (PtdSer), phosphatidylglycerol (PtdGro), and phosphatidic acid (PtdOH) (2, 11). Tachyzoites can produce these lipids using precursors imported from their extracellular and/or intracellular environment (7, 9, 12–15). PtdThr is an exceptional coccidian-specific lipid identified only in *Toxoplasma* and *Eimeria* (2, 11). A natural analog of otherwise-universal PtdSer, PtdThr has evolved to regulate the calcium-dependent gliding motility and subsequent egress-invasion events during the lytic cycle of *T. gondii*. Intriguingly, PtdThr is more abundant than PtdSer in *Toxoplasma* tachyzoites and *Eimeria* sporozoites (2, 11). The enzymes underlying the synthesis of these lipids, PtdThr synthase (PTS) and PtdSer synthase (PSS), are located in the endoplasmic reticulum of both parasites (2, 11). The PTS originated from the base-exchange type PSS and it can utilize serine and threonine as substrates. While PTS has been studied for its functional expression and physiological relevance in *T. gondii*, PSS still needs to be investigated, motivating us to undertake this study.

PtdSer in mammalian cells is a key phospholipid, contributing to the structural integrity of bio-membranes, protein sorting, and secretion (16, 17). It is decarboxylated to PtdEtn, which in turn can be methylated to produce PtdCho and thus serves as a precursor for the synthesis of major phospholipids (18, 19). Conversely, PtdEtn and PtdCho serve as donor lipids to produce PtdSer in the endoplasmic reticulum of mammalian cells via the base-exchange reaction catalyzed by PtdSer synthase (20, 21)). This enzyme, present in the endoplasmic reticulum, catalyzes the choline or ethanolamine moiety exchange in PtdCho or PtdEtn, respectively, for the serine head group to produce PtdSer. In yeast and bacterial cells, PtdSer is made by a mechanistically different enzyme utilizing CDP-DAG and serine (22). The subcellular roles of PtdSer depend primarily on its acidic nature and negative charge that can impact lipid-protein binding, calcium homeostasis, lipid asymmetry, and membrane potential (16, 21). Our previous work in *T. gondii* demonstrated that tachyzoites could synthesize PtdSer, mostly converted to PtdEtn; however, the latter lipid is not methylated to produce PtdCho (9, 23). We recently reported the flipping/uptake of exogenous PtdSer driven by a P4-type ATPase (P4-ATPase1) residing in the apical plasma membrane of tachyzoites (6, 24). Deletion of the *P4-ATPase1* gene or conditional knockdown of its partner protein LEM1 (Ligand Effector Module 1) in tachyzoites impairs the locomotion, egress, and invasion of mutants, indicating a specialized role of PtdSer during the lytic cycle. Notably, a deletion of PtdThr synthase also culminates in a similar phenotype (2, 11). Here, we show that PSS is a base-exchange-type enzyme contributing to the biogenesis of PtdSer and eventually of PtdEtn via lipid decarboxylation. It is essential for tachyzoite survival in human host cells, and its conditional depletion disrupts several lipids.

## Materials and Methods

### Biological reagents and resources

The RHΔ*ku80*Δ*hxgprt* (25), RHΔ*ku80*Δ*hxgprt*-Tir1 (26) and RHΔ*ku80*Δ*hxgprt*-DiCre (27, 28) strains were offered by Vern Carruthers (University of Michigan), David Sibley (Washington State University) and Moritz Treeck (The Francis Crick Institute) respectively. The PSS-2HA-DD and PSS-3FLAG-DiCre strains were made using the *pLIC-DHFR-TS-2HA-DD* and *pG140* plasmids donated by Markus Meissner (Ludwig-Maximilian University). The *pLinker-AID-3HA-DHFR-TS* plasmid provided by David Sibley (Washington State University) was deployed to construct the PSS-AID-3HA strain. Antibodies against *Tg*Hsp90, *Tg*Gap45, *Tg*Actin and *Tg*IMC3 proteins were given by Sergio Angel (IIB-INTECH), Dominique Soldati-Favre (University of Geneva) and Marc-Jan Gubbels (Boston CollegeA) respectively. Anti-*Tg*Sag1 and anti-FLAG antibodies were purchased from Life Technologies. Other primary antibodies recognizing the HA and YFP epitopes were obtained from MLB and Abcam. Corresponding secondary antibodies (Alexa488, Alexa594) were procured from Invitrogen, and immunoblot dyes (IRDye 680RD, IRDye 800RD) were from LI-COR Biosciences GmbH. [^14^C_3_]-L-Serine was procured from Sigma-Aldrich. The DNA oligonucleotides for molecular cloning and transgenic work (Supplemental Table S1) were obtained from Life Technologies.

### Parasite and host cell culture

Human foreskin fibroblast (HFF) cells were cultured in Dulbecco’s Modified Eagle Medium (DMEM) containing glucose (4.5 g/L), 1 mM sodium pyruvate, 2 mM glutamine, 100 μM MEM-non-essential amino acids (glycine, alanine, asparagine, aspartic acid, glutamic acid, proline, and serine), 100 U/ml penicillin,100 μg/ml streptomycin, and 10% fetal calf serum. Cells were incubated in a humidified incubator (37°C, 5% CO_2_, pH = 7.4), harvested by trypsinization, and seeded into flasks, plates, or dishes for assays. Tachyzoites were maintained by infecting confluent HFF cells (multiplicity of infection, 2–3) and passaged every two days. For phenotypic assays, parasitized host cells were scraped 40–44 h post-infection, and tachyzoites were mechanically released by squirting through 21 and 23-gauge syringes. All biochemical analyses deployed freshly-released tachyzoites washed 3x with phosphate-buffered saline (PBS, 400*g*, 10 min, 4°C). Per the experimental requirement, parasites were counted and used directly or stored at −80°C for later usage.

### Molecular cloning

RNA was isolated from extracellular parasites using a commercial kit from Jena Biosciences (Germany) and reverse transcribed into first-strand cDNA for cloning a full-length open reading frame of PSS. cDNA and gDNA were prepared using commercial kits (Life Technologies and Jena Biosciences, Germany). *Escherichia coli* (XL-1b strain) was deployed for molecular cloning and plasmid amplification. Homologous recombination-mediated genomic insertion of the AID-DHFR-TS cassette in the *Tg*PSS-AID-3HA mutant was assisted by a CRISPR plasmid, which was prepared using the Q5-site mutagenesis kit (New England Biolabs, USA) and appropriate primers (Supplemental Table S1). To engineer the PSS-3FLAG-DiCre strain, 5′ and 3′UTR of PSS (~1 kb each) were amplified using the Phanta Max Fidelity DNA polymerase (Vazyme) and cloned into *pG140* plasmid (primers in Supplemental Table S1). The amplicons were cloned into the plasmid using specific enzymes and T4 ligase (New England Biolabs).

### Making of transgenic parasites

The PSS-AID-3HA strain was generated by transfecting a CRISPR construct expressing PSS-specific *sg*RNA (*pSAG1-CAS9-U6-sgPSS*) along with a homology donor amplicon amplified from the *pLinker-AID-HA-DHFR-TS* plasmid as the template (ratio, 7:1) into tachyzoites (~10^7^) of the RHΔ*ku80*Δ*hxgprt*-Tir1 strain. Parasites were electroporated with DNA in filter-sterilized Cytomix (120 mM KCl, 0.15 mM CaCl_2_, 10 mM K_2_HPO_4_/KH_2_PO_4_, 25 mM HEPES, 2 mM EGTA, 5 mM MgCl_2_ supplemented with fresh 5 mM glutathione and 5 mM ATP; pH 7.6) using a BTX instrument (2 kV, 50 Ω, 25 μF, 250 μs). Transgenic parasites were selected by pyrimethamine (1 μM) for the expression of drug-resistant DHFR-TS (29). Tachyzoites surviving the drug selection were cloned by limiting dilution, and individual clones were screened by PCR for the events of 5′ and 3′ crossover at the PSS locus (primers in Supplemental Table S1). In parallel, clones were examined for the expression and regulation of PSS-AID-3HA by immunostaining. The eventual parasite clones expressed *Tg*PSS in an IAA-dependent manner under the control of its native promoter and *Tg*GRA1-3′UTR.

Constructing the PSS-3FLAG-DiCre mutant required successive cloning of 3FLAG-tagged ORF of PSS, 5′UTR, and 3′UTR in the *pG140* vector at the *Apa*I/*EcoR*I, *EcoR*V/*Pac*I, and *Sac*I sites, respectively. The *Apa*I-linearized construct was transfected into the RHΔ*ku80*Δ*hxgprt*-DiCre strain. Tachyzoites were selected by mycophenolic acid (25 μg/ml) and xanthine (50 μg/ml) for HXGPRT (30), cloned and screened by PCR and immunostaining. The PSS-3FLAG_floxed_ strain expressed the *lox*P-flanked *Tg*PSS-3FLAG regulated by the native promoter and the DHFR-TS-3′UTR. *Cre*-mediated deletion of PSS-3FLAG was achieved by adding rapamycin (50 nM) to the parasite culture. Excision of PSS-3FLAG_floxed_ enabled repositioning and ensuing expression of YFP under the control of the PSS promoter. The mutants could be evaluated by the loss of the 3FLAG epitope and the appearance of the YFP signal.

The PSS-2HA-DD mutant was engineered by tagging the *PSS* gene with a C-terminal 2HA-DD (destabilization domain) in the RHΔ*ku80*Δ*hxgprt* strain. A ~1.1-kb crossover sequence targeting the 3′-end of *PSS* was amplified from parasite gDNA and cloned into the *Pac*I-digested vector (*pLIC-DHFR-TS-2HA-DD*) by ligation-independent cloning (Clontech, USA). Tachyzoites were transfected with *Nsi*I-linearized *pLIC-DHFR-TS-TgPSS-2HA-DD* construct and selected with 1 μM pyrimethamine (29). The PSS-2HA-DD strain expressed *Tg*PSS-2HA-DD under the control of endogenous promoter and *Tg*TUB-3′UTR.

### Flow cytometry and immunoblot analyses

For flow cytometry, parasites (1 × 10^6^) were suspended in 0.5 ml of PBS and transferred to the sample tubes, followed by the addition of propidium iodide (1 μg/ml). Samples were analyzed for the YFP and propidium iodide signals using BD FACS DIVA8 and BD FACS ARIA III instruments. The results obtained were evaluated using Flowjo software. To perform blotting, fresh tachyzoites (10^7^/sample) were washed with PBS, pelleted (400*g*, 10 min, 4°C), and then resuspended in the Laemmli protein-loading buffer for denaturing gel electrophoresis. Proteins were resolved by 8% SDS-PAGE and blotted onto a nitrocellulose membrane (85 mA, 120 min). The blot was treated overnight (4°C) with 5% skimmed milk in Tris-buffered solution (20 mM Trisbase, 150 mM NaCl, 0.2% Tween 20, pH 7.4). The membrane was incubated with α-HA or α-FLAG (1:1,000 mouse) and *Tg*Hsp90 (1:1,000 rabbit) antibodies for 1 h, washed 3x with Tween-buffered solution (5 min each), followed by treatment with IRDye secondary antibodies (680RD, 800CW, 1:10,000, 1 h). Proteins were visualized using a LI-COR imaging system (Li-COR Biosciences).

### Indirect immunofluorescence imaging

Parasitized cells on coverslips were washed with PBS, followed by paraformaldehyde fixation (4%, 10 min) and neutralization (0.1 M glycine in PBS, 5 min). Cells were permeabilized (0.2% Triton X-100 in PBS, 20 min) and then treated with 2% bovine serum albumin (BSA) dissolved in PBS-detergent solution (20 min). Samples were incubated with primary antibodies (α-HA, 1:1,000; α-FLAG, 1:1,000; α-*Tg*Gap45, rabbit, 1:10,000; α-*Tg*Sag1, mouse, 1:150) for 1 h. The coverslips were washed 3x by PBS-detergent solution and incubated with Alexa488/594-conjugated antibodies (45 min), followed by 3x washing of samples. Immunostained cells were mounted in Fluoromount G containing DAPI (Southern Biotech) and stored at 4°C. Images were acquired by fluorescence microscopy (Zeiss).

### Lytic cycle assays

Standard phenotypic methods were used to determine the impact of PSS mutagenesis on the lytic cycle of tachyzoites in the presence or absence of Shield-1 (Chemin Pharma), rapamycin, or IAA, as noted in respective figure legends. Plaque assays were set up in HFF monolayers seeded in 6- or 12-well plates. HFFs were infected with 100–200 parasites/well and incubated for 7 days without perturbation. Cells were fixed with ice-cold methanol for 10 min, stained with crystal violet for 15 min, and washed with PBS. Plaques were imaged by a light microscope and scored (size, numbers) using the ImageJ software. The parasite yield was quantified by infecting HFF cells (MoI, 1 for 48 h), followed by counting of mechanically-released progeny. To determine the replication phenotype, confluent host cells on coverslips were infected (MoI, 1 for 24 h/48 h), fixed with paraformaldehyde, and stained using the assay-specific antibodies (replication, α-*Tg*Gap45 and α-HA; endodyogeny, α-*Tg*IMC3). The parasitophorous vacuoles with a variable number of tachyzoites were analyzed to assess the parasite replication. Endodyogeny was evaluated by the fraction of tachyzoites harboring IMC3-stained daughter cells.

To compute the invasion rates, HFFs were infected by tachyzoites of specified strains (MoI, 10; 1 h). Samples were fixed by paraformaldehyde (4%, 15 min) and stained with α-*Tg*Sag1 before membrane permeabilization. Coverslips were washed 3x with PBS and samples were permeabilized by 0.2% Triton X-100 in PBS (15 min). The second staining was performed with α-*Tg*Gap45. Samples were then stained by secondary antibodies (Alexa Fluor 488/594) and mounted in Fluoromount G/DAPI solution. Extracellular and intracellular parasites were distinguished by differential staining of Sag1 and Gap45 proteins. The percentages of invaded parasites (stained only with α-*Tg*Sag1) were used to compare the invasion efficiencies of the strains. Egress assay involved a similar staining process, but HFFs were infected at MoI of 1 and cultured for either 48 h (natural egress) or for 24 h (A23187-induced egress, Sigma). In this case, the percentage of extracellular/egressed parasites (stained only by α-*Tg*Sag1) was used to estimate the egress phenotype. To determine the gliding motility of tachyzoites, fresh syringe-released parasites (6 × 10^5^) were allowed to glide on BSA (0.01%)-coated coverslips in HBSS (30 min, 37°C, 5% CO_2_), fixed with paraformaldehyde and stained by α-*Tg*Sag1 and Alexa488 antibodies. The motile fraction and trail length were quantified using the ImageJ software.

### Isotope labeling

Lipid precursor labeling of extracellular tachyzoites (1 × 10^8^/sample) with [^3^H]-ethanolamine or [^3^H]-choline (10 μCi, 25 μM) in the presence or absence of *S*-adenosylmethionine (1 mM) was performed for 4 h at 37°C, as described previously (9). Lipids isolated from the radiolabeled tachyzoites were washed 3x with 2.1 ml of methanol:PBS: chloroform (1:0.9:0.15, v/v), and the chloroform phase with lipids was recovered. Lipids were analyzed by TLC using silica gel H plates and chloroform:methanol:2-propanol: KCl (0.25%): triethylamine (90:28:75:18:54, v/v). Phospholipids were visualized by iodine vapor or 8-anilino-1-naphthalene sulfonic acid (0.2%, w/v) and identified by their co-migration with standards. For lipidomics of ^13^C_3_-serine-labeled PSS-AID-3HA, syringe-released parasites (2 × 10^7^/sample) pre-cultured in ± 300 μM IAA (48 h) were washed 2x with PBS, filtered (5 μm) and incubated for 6 h at 37°C with 1 μM ^13^C_3_-L-serine (Sigma-Aldrich). Tachyzoites were pelleted and stored at −80°C until lipidomic analysis.

### Lipidomic analysis

Tachyzoites (1 × 10^7^/sample) grown in the absence or presence of 300 μM IAA were collected, washed 2x with PBS, and filtered (5 μm). Lipids were isolated from the parasite pellets, which were suspended in 25 μl of ammonium formate, followed by the addition of 100 μl chloroform-methanol (1:1). Samples were allowed to stand for 30 min on ice, and precipitates were removed (4°C, 10,000*g*). Of the lipid-containing supernatant, 10 μl was injected into the Acuity BEH C18 UPLC column (2.1 × 100 mm, 1.7 μ, Waters) maintained at 60°C. Lipid species were separated by a gradient of methanol and acetonitrile in water, containing 10 mM ammonium formate. The gradient elution, at a flow rate 420 of 600 μl/min, was programmed as follows (time in min, % of B): (0, 12.5), (7.5, 100), (14, 100), (14.1, 12.5), (17, 12.5). The effluent was subjected to heated electrospray ionization in the negative mode in a Sciex X500R QToF instrument (Sciex). Data-dependent MS2 spectra were collected (MS1 range 400–1,050 amu, MS2 range 50–1,050 amu) with an accumulation time of 150 ms, collision energy of 35V, and a CE spread of 15V. Data were processed using the Analytics module of SciexOS, MSDial v4.92, and R v4.1.2 (31, 32).

The inclusion of stable isotopes in glycerophospholipids species was analyzed by integration of the [M-H] - (unlabeled lipid) and [M-H+2] - ([^13^C_2_]-labeled lipid). The intensity of the labeled lipid was corrected by a binomial distribution calculation of the natural occurrence of [^13^C_2_] lipid based on the abundance of the unlabeled lipid.

### Statistics and reproducibility

Unless specified otherwise, all graphs display the mean values of 3–5 assays with the standard error of the mean (SEM) and statistical significance tested by Student’s *t* test (*P ≤ 0.05; **P ≤ 0.01; ***P ≤ 0.001; ****, and *P* ≤ 0.0001). Statistical analysis, shown in figures where applicable, was performed using the GraphPad Prism. Images are representative of multiple assays.

## Results

### Tachyzoites express a base-exchange-type PSS in the endoplasmic reticulum, contributing to PtdSer and PtdEtn synthesis

**Fig. 1:**
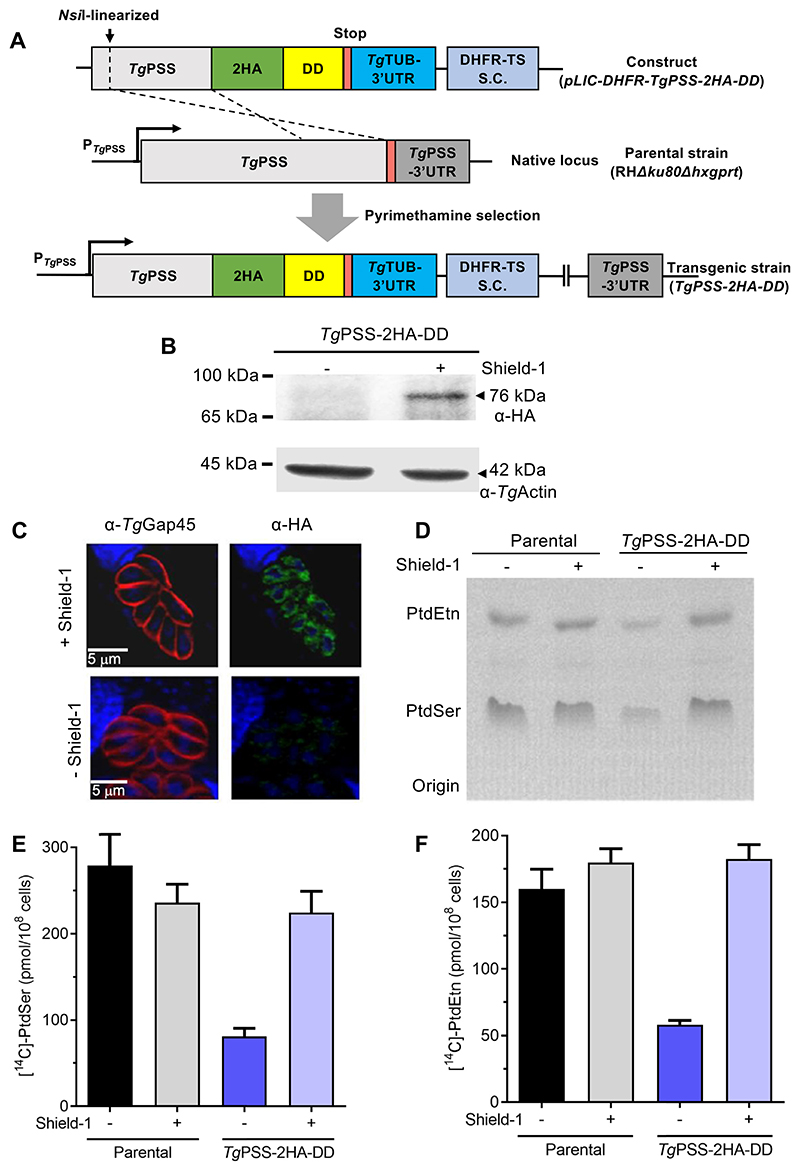
Conditional destabilization of PSS impairs PtdSer and PtdEtn synthesis. A: Schematics for C-terminal tagging of PSS with 2x Hemagglutinin-destabilization domain (2HA-DD) in tachyzoites of the RH*Δku80Δhxgprt* strain. S.C., selection cassette. B: Immunoblot showing the integrity and conditional regulation of *Tg*PSS-2HA-DD protein. Fresh extracellular parasites released from untreated or Shield-1-treated cultures (0.5 μM; MoI, 3; 44 h) were analyzed by immunoblotting using α-HA and α-actin antibodies. C: Immuno-fluorescent localization and regulation of PSS by Shield-1. Parasitized HFF cells cultured (MoI, 2; 44 h) in the absence or presence of 0.5 μM Shield-1 were stained by α-HA and α-Gap45 antibodies. DAPI shows the parasite and host-cell nuclei. D: Autoradiograph of TLC-resolved phospholipids after radiolabeling of fresh tachyzoites (5 × 10^7^/reaction) with [^14^C]-serine (2 μCi, 2 h, 37°C). Specified strains were pre-grown without or with 0.5 μM Shield-1 (MoI, 3; 44 h), followed by serine labeling, lipid isolation, and thin layer chromatography in chloroform/ethanol/water/triethylamine solvent (30/35/7/35, v/v). Individual phospholipid bands were identified by co-migration with authentic standards. E and F: Radiolabeled phospholipid bands were scraped off the plate and subjected to scintillation counting to determine the incorporation of [^14^C] in PtdSer and PtdEtn (n = 3 experiments, means ± SEM).

Our earlier work has partly characterized *Tg*PSS (TGGT1_261480) by expression in *E. coli* and subcellular localization in *T. gondii* (2). The genome-wide CRISPR mutagenesis score for *Tg*PSS is −4.8, indicating its essential function during the lytic cycle (33). Our phylogenetic analysis showed PSS clusters with the base-exchange-type orthologs, while CDP-DAG-dependent PSS proteins, and PTS-related proteins form divergent clades (Supplemental Fig. S1A). PSS harbors archetypical transmembrane domains and conserved residues needed for the base-exchange catalysis (Supplemental Fig. S1B). Besides, superimposition of the *Tg*PSS and *Hs*PSS2 domains revealed significant conservation between the catalytic residues and pockets (Supplemental Fig. S2A). These data resonate with PtdEtn-dependent PtdSer synthesis activity observed in parasite lysate (Supplemental Fig. S2B). Our attempts to purify PSS for catalytic evaluation were futile; hence, we examined its functional contribution to phospholipid synthesis directly in *T. gondii* by fusing the native PSS with a C-terminal 2HA-DD tag by 3′-insertional genomic tagging (Fig. 1A). This system allows conditional stabilization by reversible binding of a synthetic ligand (Shield-1) to an otherwise-unstable protein fused to a destabilization domain (ddFKBP) in tachyzoites (34).

Successful tagging of PSS in the RHΔ*ku80*Δ*hxgprt* tachyzoites was confirmed by immunoblot analysis, revealing a band of 76-kDa equivalent to the PSS-2HA-DD protein. Its endogenous expression could be downregulated after removing Shield-1 from the parasite culture (Fig. 1B). The fusion protein localized in the perinuclear region signifying the ER network in a ligand-dependent manner (Fig. 1C). To assess whether PSS can catalyze PtdSer synthesis, we incubated the parental and mutant strains with [^14^C]-serine (Fig. 1D–F). Tachyzoites were either untreated or pre-cultured with Shield-1 to determine the catalytic specificity of phospholipid synthesis (Fig. 1D–F). X-ray autoradiography of the total parasite lipids resolved by thin-layer chromatography displayed bands matching PtdSer and its decarboxylation product PtdEtn (Fig. 1D). We quantified radiolabeled lipids for the inclusion of [^14^C]-serine by scintillation counting (Fig. 1E, F) and recorded >60% decline in the synthesis of PtdSer in PSS-depleted (-Shield-1) strain compared to ligand-treated mutant (+Shield-1). In accord, the labeling of [^14^C]-PtdSer-derived PtdEtn was also reduced. By contrast, synthesis of both lipids was unaffected in the parental strain irrespective of compound treatment.

A major fraction of *de novo*-synthesized PtdSer was converted to PtdEtn, suggesting a role of PSS in its biogenesis. Consistent with previous studies (9, 23), no 0.5 μM Shield-1 were stained by α-HA and α-Gap45 antibodies. DAPI shows the parasite and host-cell nuclei. D: Autoradiograph of radiolabeling of PtdCho with [^14^C]-serine was detected, confirming the lack of a PtdEtn methyltransferase in tachyzoites. In extended work, we labeled extracellular parasites with [^14^C]-serine or [^14^C]-ethanolamine in the absence or presence of S-adenosylmethionine, which serves as a methyl donor for lipid methylation. As expected, radioactive serine was incorporated in PtdSer, PtdEtn, and, to some extent, in sphingolipids (Supplemental Fig. S3A), while ethanolamine was incorporated mainly in PtdEtn (Supplemental Fig. S3B). None of the samples displayed PtdCho synthesis, even with exogenously-added S-adenosylmethionine in the parasite medium, confirming that PtdEtn is not methylated to produce PtdCho in tachyzoites.

### PSS is essential for the parasite survival in human host cells

We next tested the importance of the PSS enzyme for the lytic cycle by plaque assays using the PSS-2HA-DD mutant (± Shield-1, Supplemental Fig. S4A). Unexpectedly, despite the effective down-regulation of PSS in the absence of Shield-1 (Fig. 1B, C), we did not observe a reduction in the plaque area after 7 days of incubation (Supplemental Fig. S4A). In the replication assay (40 h post-infection), however, we witnessed a higher fraction of smaller vacuoles harboring fewer parasites in PSS-depleted cultures (-Shield-1) than the control samples (+Shield-1) (Supplemental Fig. S4B). Such a weak phenotype is probably due to the residual activity of the PSS enzyme (Fig. 1D–F). Quantitative analysis of the PSS-2HA-DD mutant’s lipids resolved by TLC revealed a lower level of PtdSer (co-migrating with PtdThr), while PtdEtn was unaltered (Supplemental Fig. S4C). Given the unexpected phenotype, we did not deploy the PSS-2HA-DD strain for further work and instead engineered another strain based on rapamycin-induced dimerizable *Cre* recombinase (DiCre) (28).

**Fig. 2:**
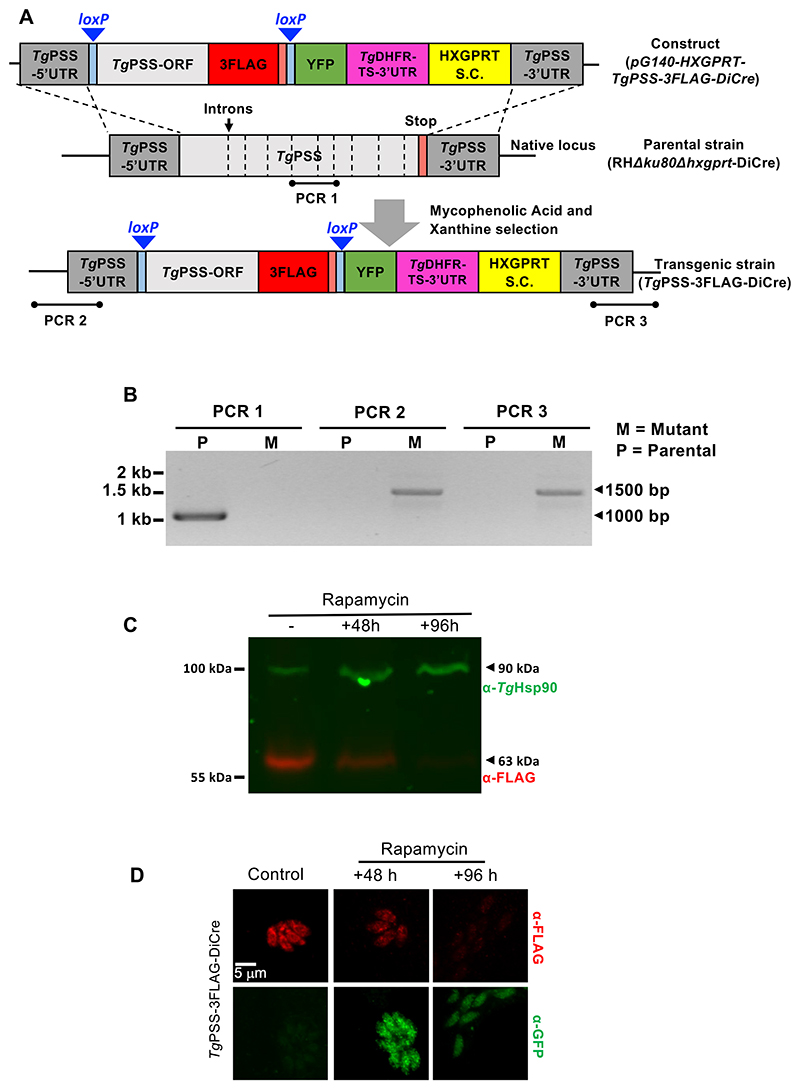
Engineering of a rapamycin-induced dimerizable *Cre*-mediated *Tg*PSS mutant. A: illustration depicting the DiCre-mediated conditional knockout of PSS in the PSS-3FLAG-DiCre strain. The native locus of PSS was substituted by a donor cassette expressing *loxP*-flanked PSS-3FLAG under the control of its 5′ and 3′UTR, and containing the HXGPRT selection cassette (S.C.) in the RH*Δku80Δhxgprt*-DiCre strain. The PSS-3FLAG-DiCre mutant allowed functional activation of a dimerizable *Cre* recombinase by rapamycin, leading to the deletion of floxed PSS-3FLAG and repositioning (expression) of YFP. B: genomic PCR screening confirming the occurrence of 5′ and 3′-crossover events, leading to the insertion of PSS-3FLAG and HXGPRT cassettes at the *PSS locus* (M, mutant; P, parental). PCR1 with intron-specific primers (dashed lines in *panel A*) shows the loss of the gene *locus* in the PSS-3FLAG-DiCre mutant. C: immunoblot revealing the integrity of PSS-3FLAG and its regulation by rapamycin. The PSS-3FLAG-DiCre strain was cultured without or with 50 nM rapamycin for 48 h or 96 h, followed by protein extraction and immunoblotting (5 × 10^6^ parasites/sample). α-FLAG staining visualizes *Tg*PSS, while α-*Tg*Hsp90 serves as a protein loading control. D: images depicting the PSS-3FLAG expression in the parasite ER (Control, -rapamycin) and appearance of YFP after rapamycin exposure (50 nM). Intracellular tachyzoites (−/+ rapamycin) were stained by α-FLAG and α-GFP antibodies.

The DiCre system is based on site-specific recombination using dimerized *Cre* recombinase. *Cre* recognizes and catalyzes the excision of DNA flanked by two identical sequences known as *loxP* sites (Fig. 2A). The recipient parasite strain (RHΔ*ku80*Δ*hxgprt*-DiCre) expressed two inactive fragments of *Cre* fused to rapamycin-binding proteins FRB and FKBP, respectively. The addition of rapamycin results in the reconstitution of the functional enzyme and excision of the *loxP*-flanked (floxed) gene of interest. The PSS gene (5′UTR + coding region + 3′UTR) was replaced by a homology donor amplicon, harboring a floxed PSS-3FLAG open reading frame and HXGPRT selection marker. This transgenic strategy enabled rapamycin-induced *Cre*-mediated deletion of the floxed PSS-3FLAG while repositioning YFP under the control of the PSS promoter. The eventual mutants could then be identified by the loss of FLAG signal concurrent with the appearance of YFP (Supplemental Fig. S5A, B).

**Fig. 3:**
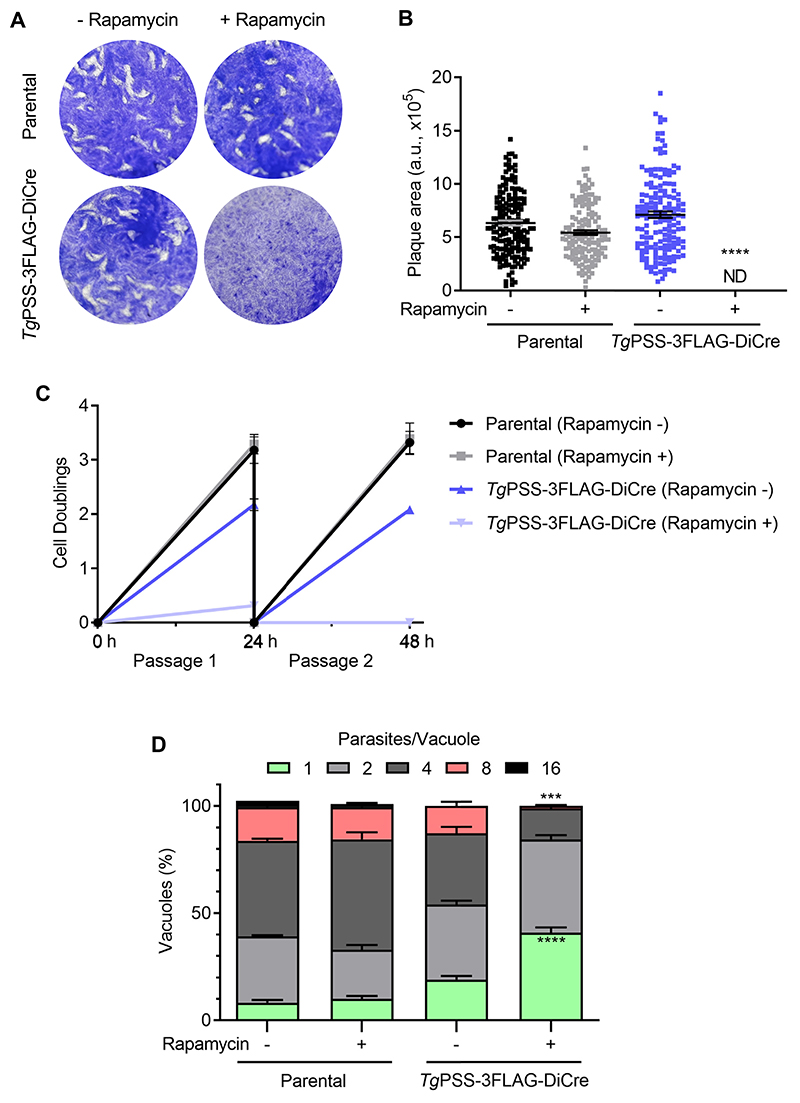
PSS is required for the tachyzoite survival. A and B: Evaluation of parasite growth by plaque assays using the PSS-3FLAG-DiCre and parental strains (−/+ 50 nM rapamycin). Crystal violet-strained images in *panel A* show plaques (white areas) formed by focal lysis of HFF monolayers (blue). *Panel B* shows the ImageJ-quantified area of about 100 plaques for each strain from 3 assays in arbitrary units (means ± SEM) (ND, not detectable). C: The parasite yield of indicated strains (−/+ 50 nM rapamycin), as determined by successive culture in HFFs (MoI, 1; n = 5 experiments, means ± SEM). D: Fractional distribution of parasitophorous vacuoles harboring a variable number of tachyzoites (1–16) after culturing in the absence or presence of 50 nM rapamycin for 24 h (MoI, 1). The replication phenotype of the two strains was evaluated by counting *Tg*Gap45-stained parasites developing within vacuoles (n = 5 assays, means ± SEM). Statistical significance in *panel B* and *D* was tested between −/+ rapamycin conditions by Student’s *t* test (***P ≤ 0.001; ****P ≤ 0.0001).

Genomic screening by recombination-specific PCR identified the PSS-3FLAG_floxed_ clones of the PSS-3FLAG-DiCre mutant (Fig. 2B). Immunoblot showing a band of 63-kDa confirmed the integrity of the epitope-tagged PSS (Fig. 2C). As expected, PSS-3FLAG was present in the ER (Fig. 2D), and exposure to rapamycin for 48 h or longer ablated its expression, coinciding with the appearance of YFP signal driven by the PSS promoter. The flow cytometry analysis of the mutant showed that about 88% parasites were YFP-negative in untreated control cultures, while the number of YFP^+^ parasites increased from 12% to 53% after 48 h of rapamycin treatment, endorsing the functionality of the DiCre system (Supplemental Fig. S5A, B). The fraction of nonviable tachyzoite stained with propidium iodide (PI) was approximately 10%, irrespective of rapamycin in culture. Next, we examined the growth phenotype of the mutant by plaque and replication assays (Fig. 3). Unlike the parental strain, which was unaffected by rapamycin, plaque formation by the PSS-3FLAG-DiCre strain was abolished in its presence (Fig. 3A, B). Likewise, the PSS-knockout mutant ceased to grow in cultures after 48 h (Fig. 3C) and formed significantly smaller vacuoles harboring only 1 to 4 parasites, unlike the untreated control and parental cultures with much larger vacuoles containing 8–16 tachyzoites (Fig. 3D). Collectively, these data demonstrate an essential function of PSS for parasite growth in human host cells.

### Knockdown of PSS impairs the cell division and locomotion of *T. gondii*

**Fig. 4:**
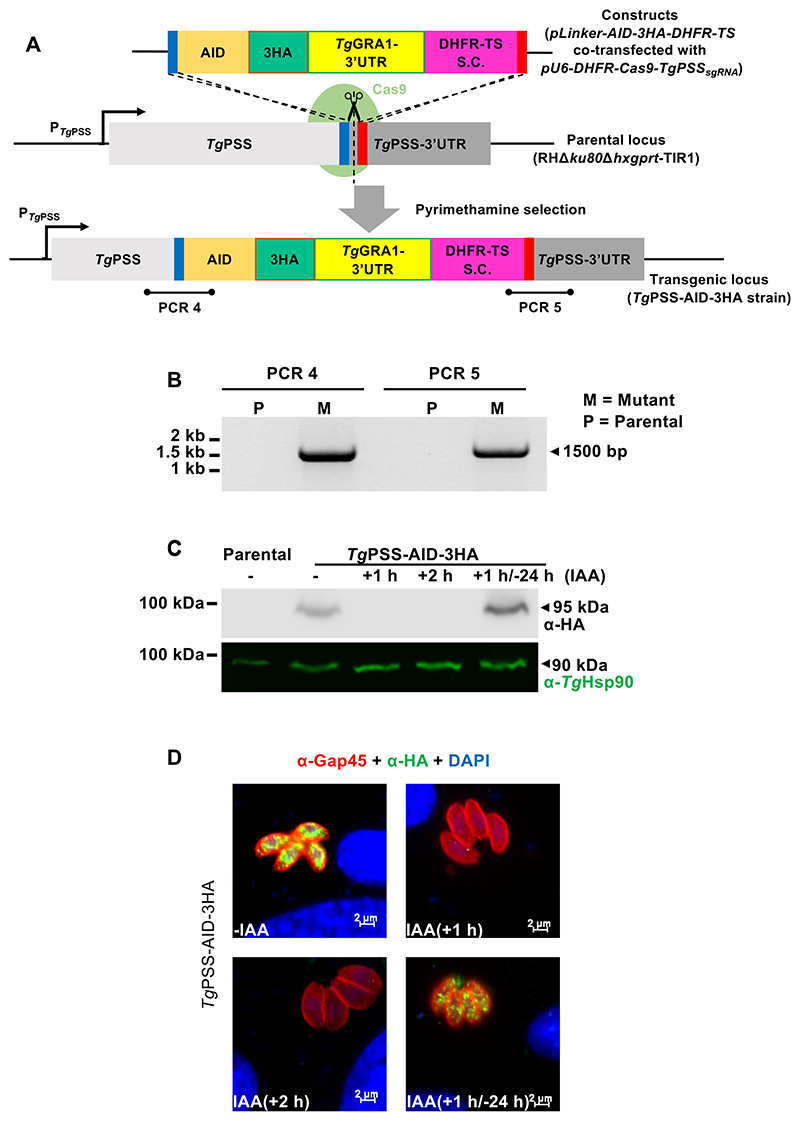
Conditional knockdown of PSS by auxin-inducible degron (AID). A: Schematic diagram of 3′-genomic tagging of PSS, resulting in auxin-regulatable expression of PSS-AID-3HA. The *Tg*PSS-AID-3HA strain was isolated by pyrimethamine selection, PCR screening, and dilution plating. S.C., selection cassette. B: Genomic PCR confirming the AID cassette insertion at the *PSS locus* in the *Tg*PSS-AID-3HA mutant. Screening primers are marked in *panel A*. C: Immunoblot of the parental and mutant strains cultured in the absence/presence of 500 μM IAA. Intracellular tachyzoites incubated with IAA for indicated periods were syringe-released for immunoblotting (5 × 10^6^ parasites/sample). A sample treated with IAA for 1 h and then cultured in a standard medium for 24 h was also included to test the reversible regulation of PSS-AID-3HA. Blots were probed for HA tag and *Tg*Hsp90. D: Immunofluorescent images depicting the IAA-regulation of PSS in the *Tg*PSS-AID-3HA strain. Parasitized cells exposed to 500 μM IAA (MoI, 1; 24 h) were stained to detect the HA tag, *Tg*Gap45, and cell nuclei.

While the PSS-3FLAG-DiCre mutant enabled affirmative testing of the PSS’s essentiality in tachyzoites, we could not obtain sufficient number of parasites for downstream assays. We, therefore, constructed another conditional mutant based on auxin-inducible degron (AID), which allows rapid proteasomal degradation of the AID-tagged proteins by indole-3-acetic acid (IAA) in *T. gondii* (26). Two transgenic components are needed to implement this system: a plant auxin receptor called transport inhibitor response 1 (Tir1) and a protein of interest tagged with an AID. The PSS protein was fused with an AID-3HA tag at its “C’ terminus using CRISPR-Cas9-based genomic engineering of the RHΔ*ku80*Δ*hxgprt*-Tir1 strain. A donor amplicon bearing 5′ and 3′ homology arms of PSS (≈40 bp), AID-3HA domain, and DHFR-TS selection cassette was transfected into tachyzoites along with a CRISPR plasmid encoding a gene-specific *sg*RNA (Fig. 4A). The clonal transgenic PSS-AID-3HA strain was validated by genomic PCR, immunoblot, and immunofluorescence analyses (Fig. 4B–D). The size and sequence of the PCR amplicons confirmed the occurrence of homologous crossover events at the desired locus (Fig. 4B) and immunoblot revealed a band of 95-kDa representing the PSS-AID-3HA fusion protein (Fig. 4C). Immunofluorescent images indicated a perinuclear localization of PSS (Fig. 4D). Not least, PSS expression was repressed within 1–2 h culture with IAA and reappeared upon its removal (Fig. 4C, D).

**Fig. 5:**
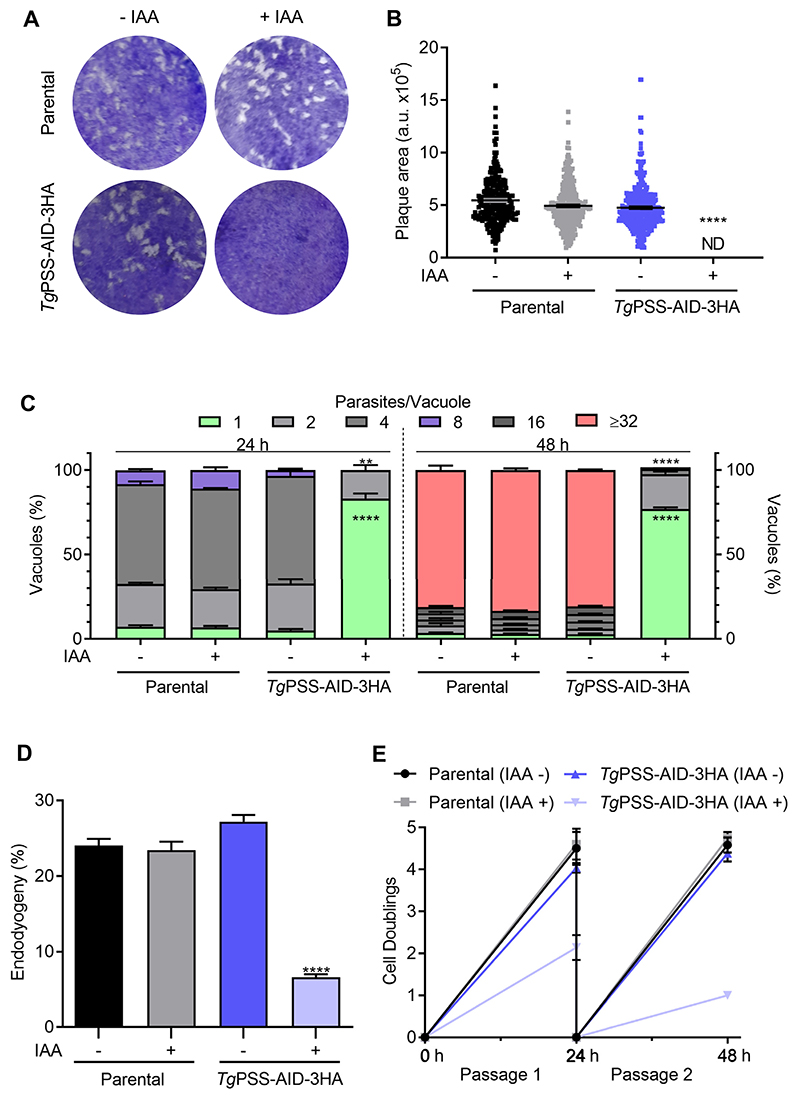
PSS depletion compromises the parasite growth due to impaired cell division. A and B: Plaque assays demonstrating the asexual growth of the *Tg*PSS-AID-3HA and parental strains (−/+ 500 μM IAA). Crystal violet-stained images indicate parasite plaques (white area) formed on host cell monolayers (blue). *Panel B* represents the size of plaques measured by ImageJ in arbitrary units (150 plaques/strain, n = 3 assays, means ± SEM). C: The replication rate of the mutant and parental strains, as judged by the fraction of vacuoles harboring a variable number of progeny (−/+ 500 μM IAA). D: Endodyogeny (daughter cell budding) in parasite strains immunostained by α-IMC-3 antibody. E: Yield assay scored by sequential propagation of specified strains (MoI, 1; 500 μM IAA). *Panels C*–*E* display the results of 5 assays (means ± SEM). Statistical significance in *panels B*–*D* was tested between −/+ IAA conditions by Student’s *t* test (**P ≤ 0.01; ****P ≤ 0.0001).

**Fig. 6:**
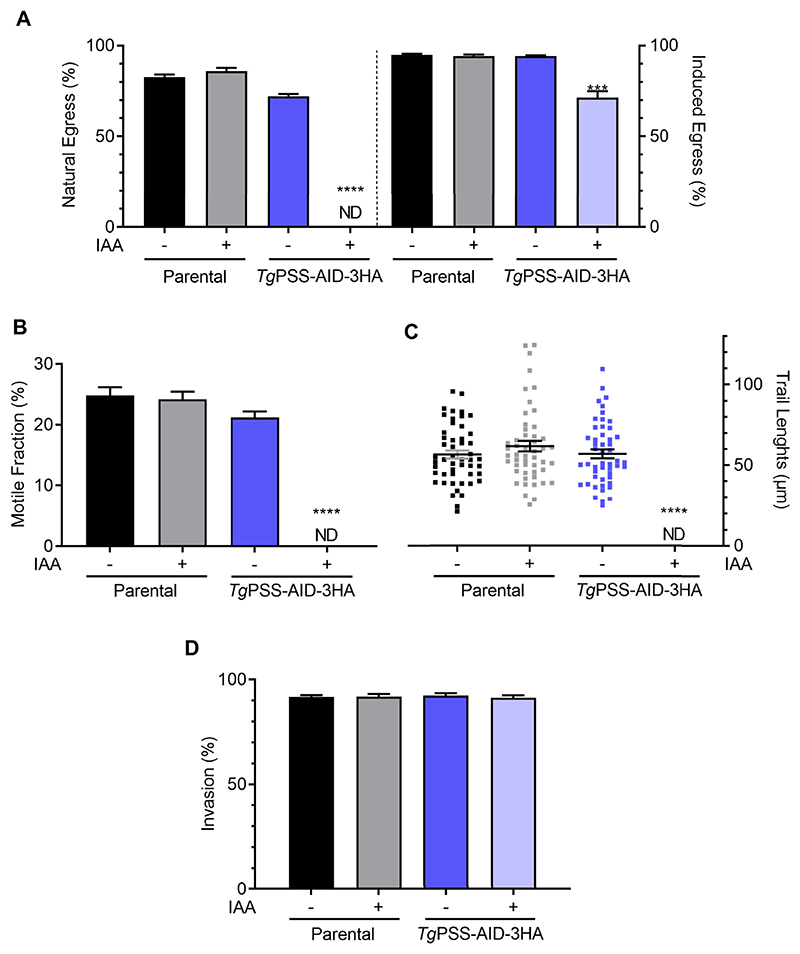
PSS-depleted parasites are impaired in gliding motility and natural egress but show a normal invasion phenotype. A: Egress of the parental and *Tg*PSS-AID-3HA strains (−/+ 500 μM IAA). Induced egress was measured after exposure to A23187 ionophore for 5 min and 500 vacuoles were analyzed to score the phenotype. B and C: Gliding motility of tachyzoites, as deduced by motile fraction and trail lengths after α-*Tg*Sag1 staining. About 500 parasites of each strain were analyzed for the motile fraction, and 50 trails were measured using ImageJ. D: Invasion efficiency of designated strains (based on 500 events). *Panel A*–*D* show the data with means ± SEM of 5 assays (ND, not detectable). Statistical significance in *panels A*–*D* was tested between −/+ IAA conditions by Student’s *t* test (***P ≤ 0.001; ****P ≤ 0.0001).

Rapid and reversible regulation of PSS-AID-3HA permitted detailed phenotypic analysis of the mutant. To begin with, we evaluated the effect of PSS knock-down on the parasite fitness. In contrast to the control cultures, IAA-treated parasites showed a severe reduction in plaque size (Fig. 5A, B), indicating a compromised growth. The replication assay showed a negative impact on the number of parasites/vacuoles in early (24 h) and late (48 h) PSS-depleted cultures (Fig. 5C). These data were corroborated by scoring the daughter cell budding (endodyogeny) within dividing tachyzoites (Fig. 5D). Likewise, the yield of the IAA-exposed mutant diminished following successive passages (Fig. 5E). As expected, due to impaired replication, the natural egress was not apparent in the PSS-depleted strain after 48 h. However, Tachyzoites could exit the host cells upon induction by A23187 (Fig. 6A)—a calcium ionophore triggering the calcium-dependent gliding motility in *T. gondii* (35). Surprisingly, the IAA-cultured PSS-AID-3HA parasites displayed no measurable motility, as quantified by motile fraction and trail length (Fig. 6B, C). Ironically, however, despite defective locomotion, the PSS-depleted mutant could invade host cells normally (Fig. 6D).

### PSS depletion dysregulates several major phospholipids in tachyzoites

**Fig. 7:**
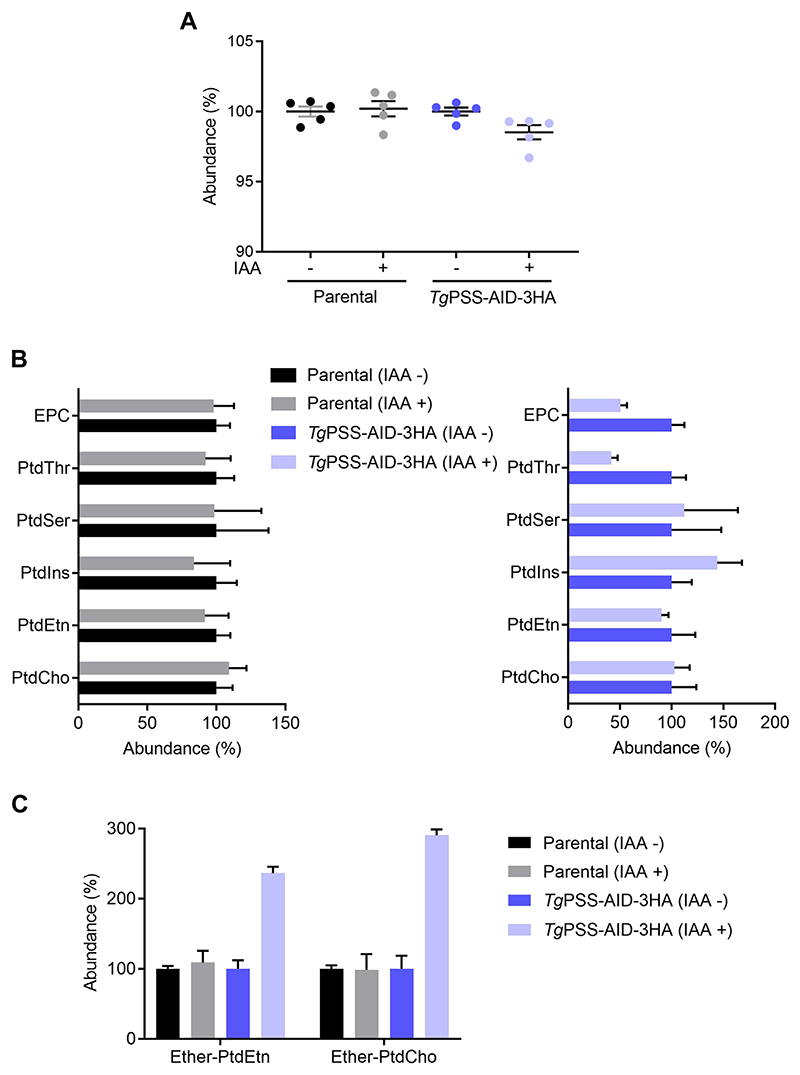
The PSS mutant shows perturbation of specific ester and ether-linked lipids. A: Phospholipid levels of the *Tg*PSS-AID-3HA and parental strains (−/+IAA). Lipids isolated from indicated parasites were quantified. B: Relative abundance of individual ester-linked phospholipids. C: Changes in ether-linked PtdEtn and PtdCho species of the PSS mutant and parental strain. Individual +IAA samples are normalized to respective -IAA samples set as 100%. *Panels A*–*C* show the data with means ± SEM of 5 experiments.

**Fig. 8:**
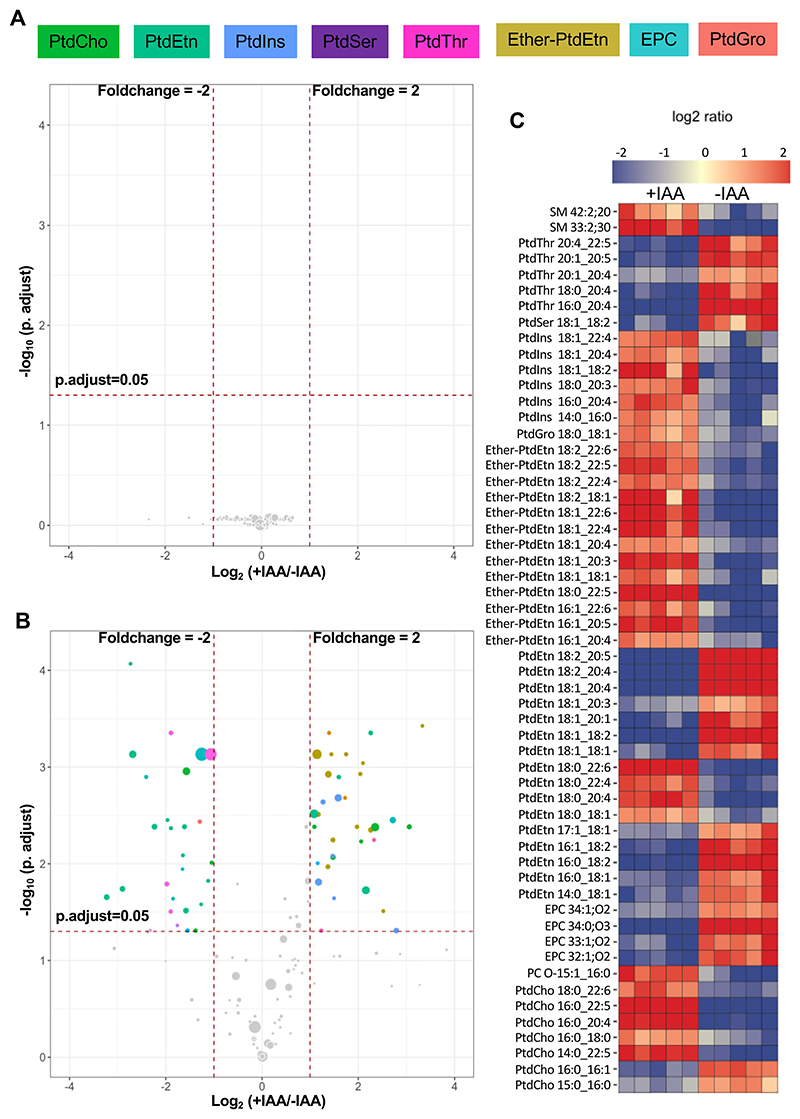
Conditional loss of PtdSer synthase modulates several phospholipid species. A and B: Volcano plots depicting changes in all detectable phospholipids upon IAA treatment of the parental and *Tg*PSS-AID-3HA strains. Thresholds of false-discovery rate (FDR)-corrected *P*-value (≤0.05) and fold-change (≥1.5) define significantly-modulated species. The horizontal dashed line represents statistical significance, and the vertical dashed lines mark the increase or decline in abundance by a factor of 1.5. Metabolite circles are scaled to abundance. Lipids qualifying the thresholds are colored according to their class, while others are indicated in gray (n = 5 assays). C: Heatmaps of the induced or repressed lipid species from *panels A* and *B*.

Lipidomic analysis of tachyzoites was undertaken to discern the role of PSS in membrane biogenesis (Figs. 7 and 8). Since the loss of PSS caused a strong growth defect and eventual death of parasites, we standardized the IAA treatment to obtain a sufficient number of viable tachyzoites for isolating lipids (Supplemental Fig. S6A). Incubation of the PSS-AID-3HA mutant with 250 μM IAA for 48 h reduced the yield by about 70% (Supplemental Fig. S6A) while allowing us to collect a viable and infective parasite population. We controlled the ‘*infective nature*’ of the IAA-grown parasites collected for lipidomics by their ability to form plaques without IAA (Supplemental Fig. S6B). Indeed, the IAA-treated PSS-AID-3HA strain was recovered after the removal of IAA. The flow cytometry analysis revealed over 85% viability of the IAA-treated PSS mutant, similar to the untreated and parental control groups (Supplemental Fig. S7A, B). Four cohorts of samples (−/+IAA parental and PSS-AID-3HA mutant) pre-controlled for viability and infectivity were subjected to lipid profiling by mass spectrometry.

We identified all major phospholipids in *T. gondii* tachyzoites, as also reported previously (2, 11) (Fig. 7). Auxin treatment of the mutant resulted in a modest decline in phospholipid abundance compared to the parental (−/+IAA) and untreated mutant samples (Fig. 7A). As expected, the major phospholipid classes showed little to no perturbation in the IAA-cultured parental strain (Fig. 7B). On the other hand, while the content of PtdCho and PtdSer was unaltered upon depletion of PSS, PtdIns increased, and PtdThr, PtdEtn, and ethanolamine-phosphorylceramide (EPC) were repressed. Our analysis also underlined a notable increase in “ether-linked” lipids, namely ether-PtdEtn and ether-PtdCho, after knockdown of PSS in the mutant (Fig. 7C). In conclusion, we observed a homeostatic perturbation of many standard (ester) and ether-phospholipids upon depletion of PSS.

### Loss of PSS causes an imbalance of selected ether and ester-linked lipid species

We next plotted the magnitude of change in all identifiable phospholipid species irrespective of their abundance as volcano plots. The data illustrates the statistical significance versus the fold-regulation upon depletion of PSS by IAA (Fig. 8). As anticipated, none of the lipid species were affected in the parental strain (≥1.5-fold, false discovery rate ≤0.05). In contrast, the PSS-AID-3HA mutant displayed modulation of multiple lipid species including PtdThr, PtdIns, PtdCho, PtdEtn, EPC, and ether-PtdEtn. A few species of PtdGro, sphingomyelin, ether-PtdCho, and PtdSer were also significantly altered (Fig. 8). As reported earlier (2, 7, 15), PtdSer species were considerably less abundant in tachyzoites, requiring in-depth peak annotation for an unambiguous detection. This provision, however, resulted in high variation and thus no statistical significance between −/+IAA samples. Nonetheless, given the focus of this work, we plotted all detectable PtdSer species irrespective of significance. As shown (Supplemental Fig. S8A), C36:3 and C34:2 were repressed in IAA-treated mutant, whereas C38:4 and C36:2 were elevated, and C34:1 was unaltered.

Moreover, several PtdSer and PtdEtn species (18:0/20:4; 18:1/18:1; 18:1/18:2; 16:0/18:1; 16:0/18:2) shared the composition of acyl chains (Supplemental Fig. S8B), likely due to PSD activity in tachyzoites (9, 23, 36). Such PtdEtn species were also reduced upon loss of PSS, suggesting their synthesis by decarboxylation of PSS-derived PtdSer.

**Fig. 9:**
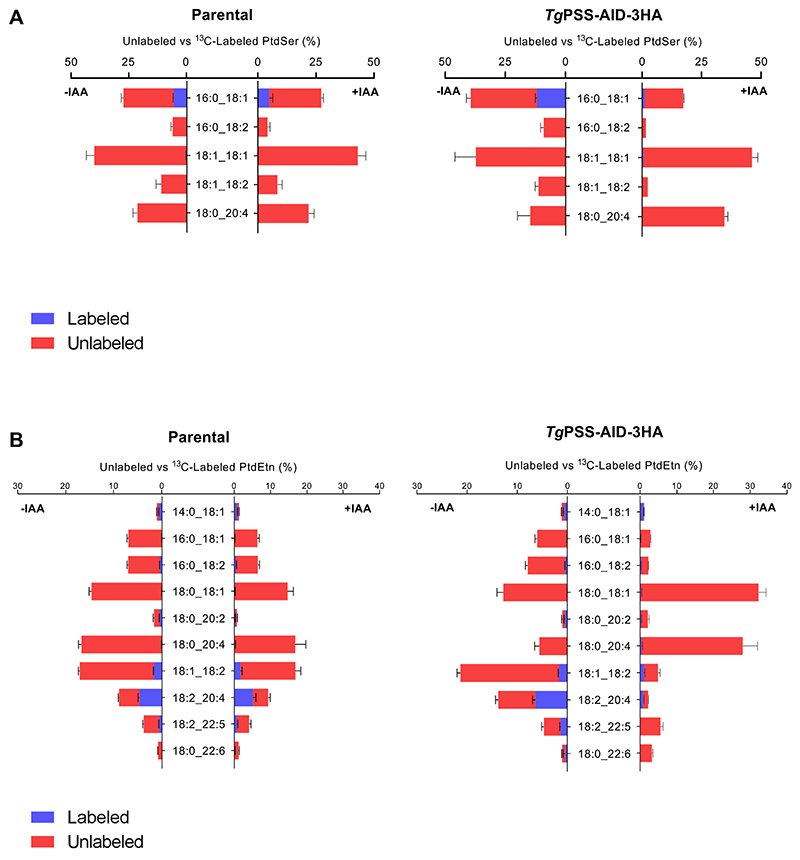
Lipidomics of [^13^C]-serine-labeled PSS-AID-3HA mutant reveals a role of PSS in the synthesis of specific PtdSer and PtdEtn species. A and B: Species of PtdSer or PtdEtn detected in tachyzoites of the PSS-AID-3HA and parental strains labeled with [^13^C]-serine in the absence or presence of IAA (n = 5 assays, means ± SEM). Fresh syringe-released parasites were incubated with the stable isotope (4 h, 37°C), followed by lipid isolation and lipidomic profiling. Graphs show the fraction of [^13^C]-labeled PtdSer (A) or PtdEtn (B) species compared to corresponding unlabeled forms. For PtdSer, labeled and unlabeled species add up to 100% in each graph, whereas for PtdEtn, only the top 10 species (amounting to >90%) are depicted for clarity. A decline in the (isotope-labeled and/or unlabeled fractions) of a lipid species in the IAA-treated mutant suggests a requirement of PSS for its synthesis. While IAA-dependent synthesis of selected PtdEtn species reflects the contribution of the PSS-PSD pathway, its unlabeled species (exclusively red-colored) are likely produced via the CDP-ethanolamine pathway.

In our final assays, we labeled syringe-released extracellular tachyzoites of the PSS-AID-3HA strain with [^13^C]-serine (−/+IAA) and analyzed the tracer inclusion in glycerophospholipids (Fig. 9A, B). Only PtdSer and PtdEtn species were labeled, and the isotope was mainly observed in the latter lipid due to high decarboxylation activity in tachyzoites (9, 23, 36). Of the five detected PtdSer species, only 16:0/18:1 showed notable ^13^C-inclusion in extracellular parasites, which was dependent on PSS expression in the mutant (Fig. 9A). As expected, some PtdEtn species (18:1/18:2, 18:2/20:4, 18:2/22:5) were also labeled in a PSS-dependent manner (Fig. 9B). These PtdEtn species declined upon IAA treatment in the PSS-AID-3HA mutant but not in the parental strain, suggesting their synthesis via the PSS-PSD pathway. The continued labeling of a few other PtdEtn species in IAA-treated culture can be attributed to compensatory (PTS-PSD) and/or alternative (via CDP-DAG-dependent PSS) pathways in tachyzoites, whereas the unlabeled PtdEtn species (only red-colored in Fig. 9B) are likely made by the CDP-ethanolamine route. In brief, the PSS-AID-3HA mutant exhibited dysregulation of many phospholipid classes, some of which are a direct consequence of PSS depletion (selected PtdEtn and PtdSer species), while others are likely due to subcellular homeostasis.

## Discussion

**Fig. 10:**
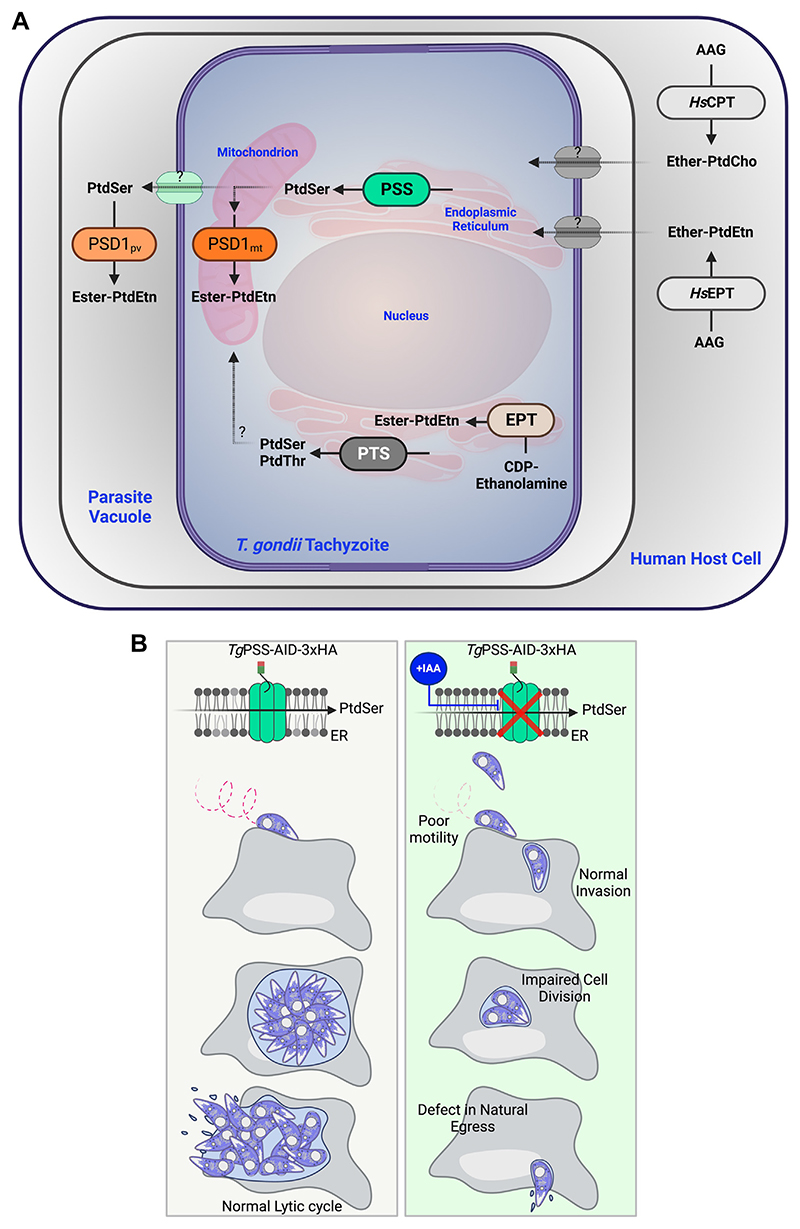
A model of PtdSer synthase mutant in tachyzoites of *T. gondii*. A: Illustration showing the synthesis and homeostatic inter-regulation of phospholipids based on this study and relevant literature. The parasite expresses a base-exchange type PtdSer synthase in the endoplasmic reticulum to synthesize PtdSer using serine and donor lipids (PtdEtn or PtdCho). A bulk of newly-made PtdSer traffics to the mitochondrion through a yet-unknown mechanism and is decarboxylated by a PtdSer decarboxylase (PSD1_mt_) to produce PtdEtn. Tachyzoites synthesize ester-linked PtdEtn species via PSD1_mt_ and the CDP-ethanolamine pathway in the ER, whereas ether-linked PtdEtn and PtdCho are salvaged from the host cell. Knockdown of PSS by indole-3-acetic acid (IAA) in the *Tg*PSS-AID-3HA strain elevates ether-linked PtdEtn and PtdCho, likely salvaged from the host cell to compensate for the decline in ester-linked lipids. PtdThr synthase (PTS) in the ER usually produces PtdThr but can catalyze the synthesis of PtdSer upon depletion of PSS. B: Model illustrating the physiological relevance of PSS, which is essential for parasite survival. Loss of PSS impairs gliding motility, cell division, and egress. The relationship between lipid modulation and phenotype of the PSS mutant is unclear.

Phospholipid synthesis versus salvage pathways in apicomplexan parasites has long been of interest to discern the network design principles of intracellular parasitism and to develop pathogen-specific therapeutics. Apicomplexan parasites harbor several parasite enzymes and transporters contributing to membrane biogenesis (11, 12, 37–40). In the context of this work, we have previously reported the occurrence of a PtdSer synthase in *T. gondii* (2), which was characterized here for its contribution to lipid synthesis, physiological relevance, and therapeutic potential. We show PSS plays a significant role in the synthesis of PtdSer, and of PtdEtn via lipid decarboxylation activity in the parasite (Fig. 10A). Additional experiments disclosed the essentiality of this enzyme for the lytic cycle and underlying events, namely cell division and gliding motility (Fig. 10B). The latter phenotype indicates a specialized functional contribution of PSS to serve the parasitic lifestyle. Conditional depletion of PSS dysregulated major ester-linked glycerophospholipids and sphingolipids, along with an increase in ether-linked PtdEtn and PtdCho species, potentially acquired from the host cell.

Glycerophospholipids have recently emerged as significant players in stage differentiation and signaling events during the intracellular development of apicomplexan parasites (2–8, 11, 41). In *T. gondii* tachyzoites, while PtdThr and PtdIns are implicated in regulating calcium signaling and homeostasis in tachyzoites (4, 7), PtdOH and PtdSer promote microneme exocytosis and gliding motility in response to calcium signaling (5, 8), The PSS-depleted mutant also suggests a role of PtdSer in regulating the locomotion of tachyzoites. A recent study deployed electron microscopy (quick-freeze-fracture replica labeling) to determine the distribution of PtdSer and PtdEtn in parasite membrane leaflets (42). The authors observed that both lipids are primarily localized in the luminal leaflet of the inner membrane complex (IMC), located just underneath the plasma membrane. The IMC harbors the actin-myosin motor machinery (glideosome), driving the parasite motility during invasion and egress events (43). We speculate that perturbation of selected PtdSer and other lipid species in the PSS-depleted strain compromises the integrity of IMC and/or micronemal secretion, which in turn, impairs the parasite motility.

The IMC originates from Golgi-derived flattened vesicles (44). Konishi *et al*. found that PtdSer is mainly confined to the cytoplasmic leaflet of the middle IMC membrane, while it was absent in the cytoplasmic leaflet of the inner IMC membrane (42). The data suggest a barrier-like mechanism preventing diffusion of PtdSer in the cytoplasmic leaflets of the two membranes. Our earlier work has reported the occurrence of five P4-type ATPases (P4-ATPases1-5), four of which are expressed at various subcellular sites in tachyzoites (24). P4-ATPase1, along with its partner subunit LEM1 (*a.k.a*. ATP2B-CDC50.4 complex), is located in the conoidal region of the parasite, where it regulates the asymmetric distribution of PtdSer and thereby facilitates the organelle fusion for micronemal exocytosis and gliding motility (5, 6, 24). Our work on P4-ATPase2 revealed its predominant expression in the IMC (24), which we believe underlies the asymmetric distribution of PtdSer in this organelle. Like P4-ATPase1, expression of P4-ATPase2 is critical, albeit not essential, for parasite growth (6). It is plausible that the physiological essentiality of PSS resonates with P4-ATPase1 and P4-ATPase2 deletion mutants, leading to defective IMC and micronemal exocytosis, which remains to be examined.

PtdThr is a structural analog of PtdSer, which complicates the interpretation of PtdSer-related works described above. Our preliminary work has revealed that tools such as PtdSer-specific biosensors (Lact-C2-GFP, (45)) and electron microscopy (42) cannot discriminate between PtdSer and PtdThr. Furthermore, albeit PtdThr can now be synthesized (46), its research-grade probes are not commercially available. We also know that while PtdSer synthase produces only PtdSer, PtdThr synthase can synthesize PtdThr and PtdSer (2). Most PtdThr species declined in the PSS mutant, whereas PtdSer species were perturbed only modestly, possibly due to the serine-exchange reaction of PTS (2) favoring the synthesis of PtdSer upon depletion of PSS. An alternative explanation for an unperturbed level of PtdSer in the mutant lies in the occurrence of yet-unknown CDP-DAG-dependent PtdSer synthase, as indicated by our earlier work (7, 47). An unambiguous assessment of the functional contribution of PtdSer and PtdThr in *T. gondii* remains challenging due to the non-availability of apposite tools. It would be imperative to devise approaches such as lipid-specific biosensors, fluorescent probes of PtdThr, isotope-labeling of PSS/PTS mutants, and additional parasite strains engineered with threonine or serine-specific PTS/PSS enzymes to differentiate the subcellular functions of both lipids.

Lipidomic analysis of the PSS mutant allowed us to compare lipid species across their classes and thereby discern the inter-regulation of phospholipid homeostasis in *T. gondii*. For instance, some PtdSer and PtdEtn species shared the acyl chains and were similarly altered, suggesting a conversion of PSS-derived selected PtdSer species into PtdEtn. Similarly, a few EPC species, probably derived from PtdEtn, were declined. By contrast, we recorded a significant rise in ether-linked PtdEtn and PtdCho species, most plausibly salvaged from the host cells, as discovered recently in a phosphoethanolamine cytidylyltransferase (ECT) mutant deficient in PtdEtn synthesis (15). Last, the upregulation of several PtdIns species upon depletion of PSS appears to balance a decline in anionic phospholipids - a reciprocal equivalent of the PtdIns synthase mutant (7). Not surprisingly, such dysregulation of phospholipids and sphingolipids culminates in a rapid death of the PSS mutant. While specific reasons underlying the essentiality of PtdSer synthase in tachyzoites merit further research, our study demonstrates the importance of PSS enzyme for the biogenesis of selected PtdSer and PtdEtn species and endorses it as a therapeutic target to treat acute toxoplasmosis.

## Supplementary Material

Supplementary Material

## Data Availability

The data generated during this study are provided in the main article and supplementary files. The source data for oligonucleotides and lipidomics can be found in the Excel files (Supplemental Tables S1 and S2). Lipidomics data have also been deposited in the Mendeley Data Repository and accessible through the link https://doi.org/10.17632/6wzfkwxhnf.1. All biological resources are available from the authors upon reasonable request.
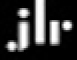

## Abbreviations

AID: auxin-induced degron
CRISPR: clustered regularly interspaced short palindromic repeat
DHFR-TS: a pyrimethamine-resistant mutant of dihydrofolate reductase-thymidylate synthase
EPC: ethanolamine phosphorylceramide
ER: endoplasmic reticulum
HFF: human foreskin fibroblast
HXGPRT: hypoxanthine-xanthine-guanine phosphoribosyl transferase
IAA: indole-3-acetic acid
PSD: phosphatidylserine decarboxylase
PSS: phosphatidylserine synthase
PtdCho: phosphatidylcholine
PtdEtn: phosphatidylethanolamine
PtdGro: phosphatidylglycerol
PtdIns: phosphatidylinositol
PtdSer: phosphatidylserine
PtdThr: phosphatidylthreonine
PTS: phosphatidylthreonine synthase
PV: parasitophorous vacuole
*sg*RNA: single guide RNA.
