# Supplementary material for: Phosphatidylserine synthase in the endoplasmic reticulum of Toxoplasma is essential for its lytic cycle in human cells
Source: J Lipid Res. Author manuscript; Available in PMC 2024 Jun 14. (PMC11166882; doi:10.1016/j.jlr.2024.100535)
Supplement: Supplementary Material [file EMS196775-supplement-Supplementary_Material.zip › 1-s2.0-S0022227524000403-mmc1.pdf]

Figure S1

A

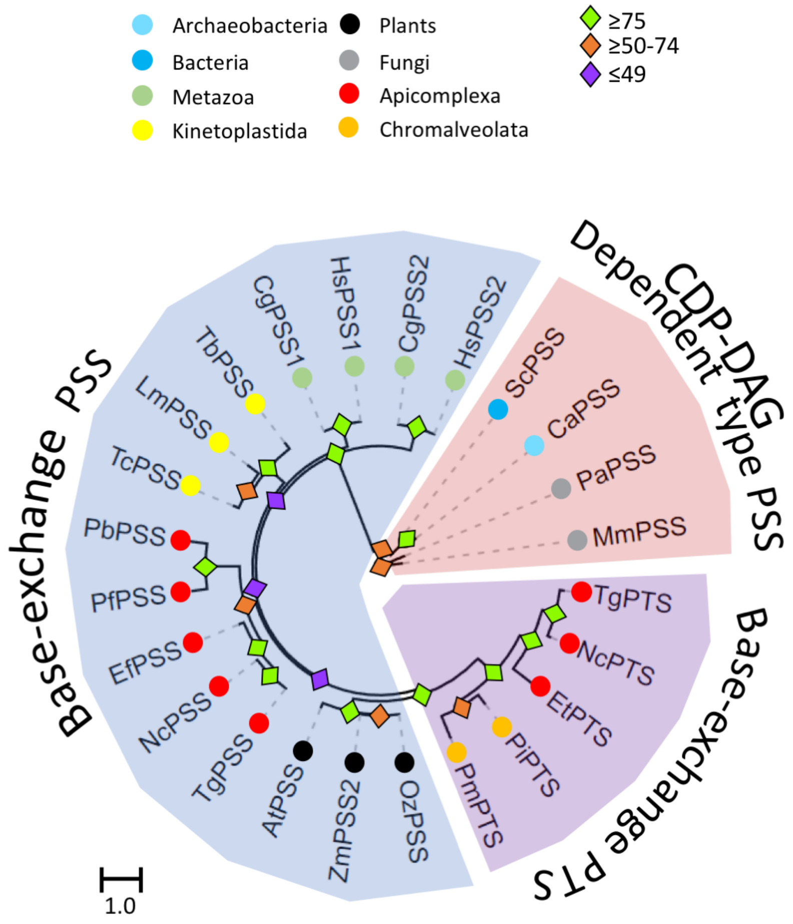

Archaeobacteria
Bacteria
Metazoa
Kinetoplastida
Plants
Fungi
Apicomplexa
Chromalveolata
≥75
≥50-74
≤49
Base-exchange PSS
CDP-DAG Dependent type PSS
Base-exchange PTS
CgPSS1
HsPSS1
CgPSS2
HsPSS2
TbPSS
LmPSS
TcPSS
PbPSS
PfPSS
EfPSS
NcPSS
TgPSS
AtPSS
ZmPSS2
OzPSS
ScPSS
CaPSS
PaPSS
MmPSS
TgPTS
NcPTS
EtPTS
PiPTS
PmPTS
1.0

B

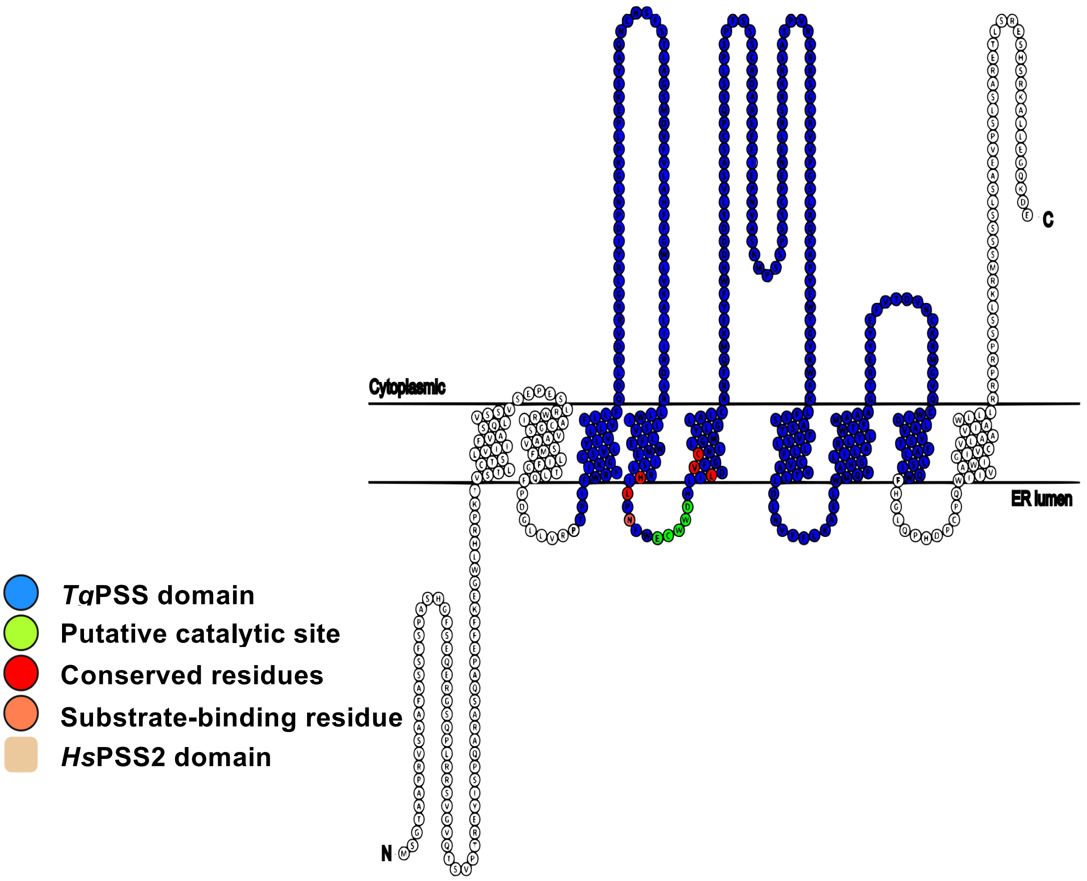

Cytoplasmic
ER lumen
N
C
TgPSS domain
Putative catalytic site
Conserved residues
Substrate-binding residue
HsPSS2 domain

Figure S2

A

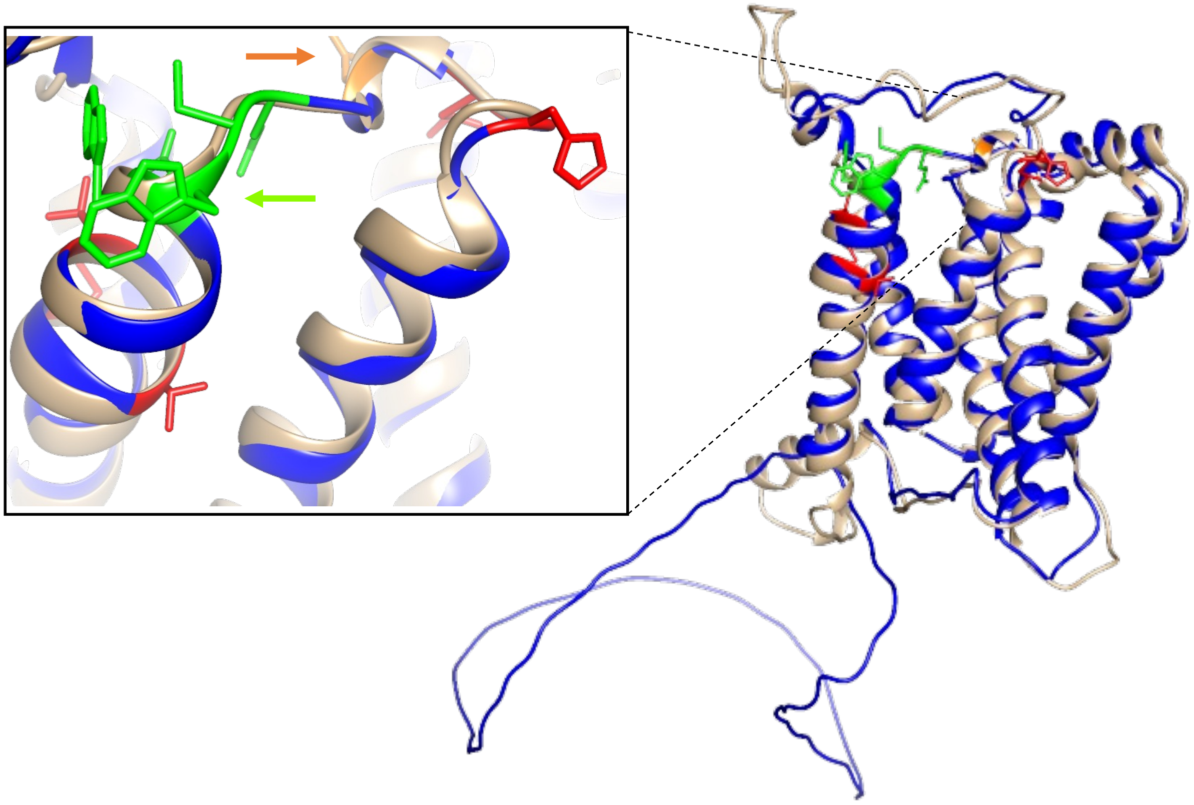

B

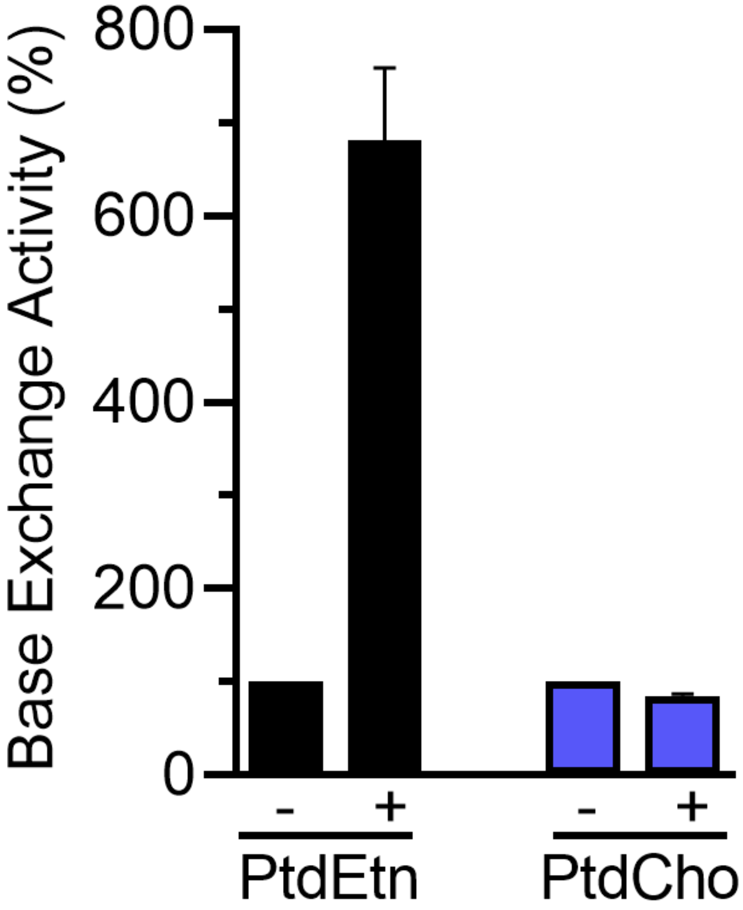

Figure S3

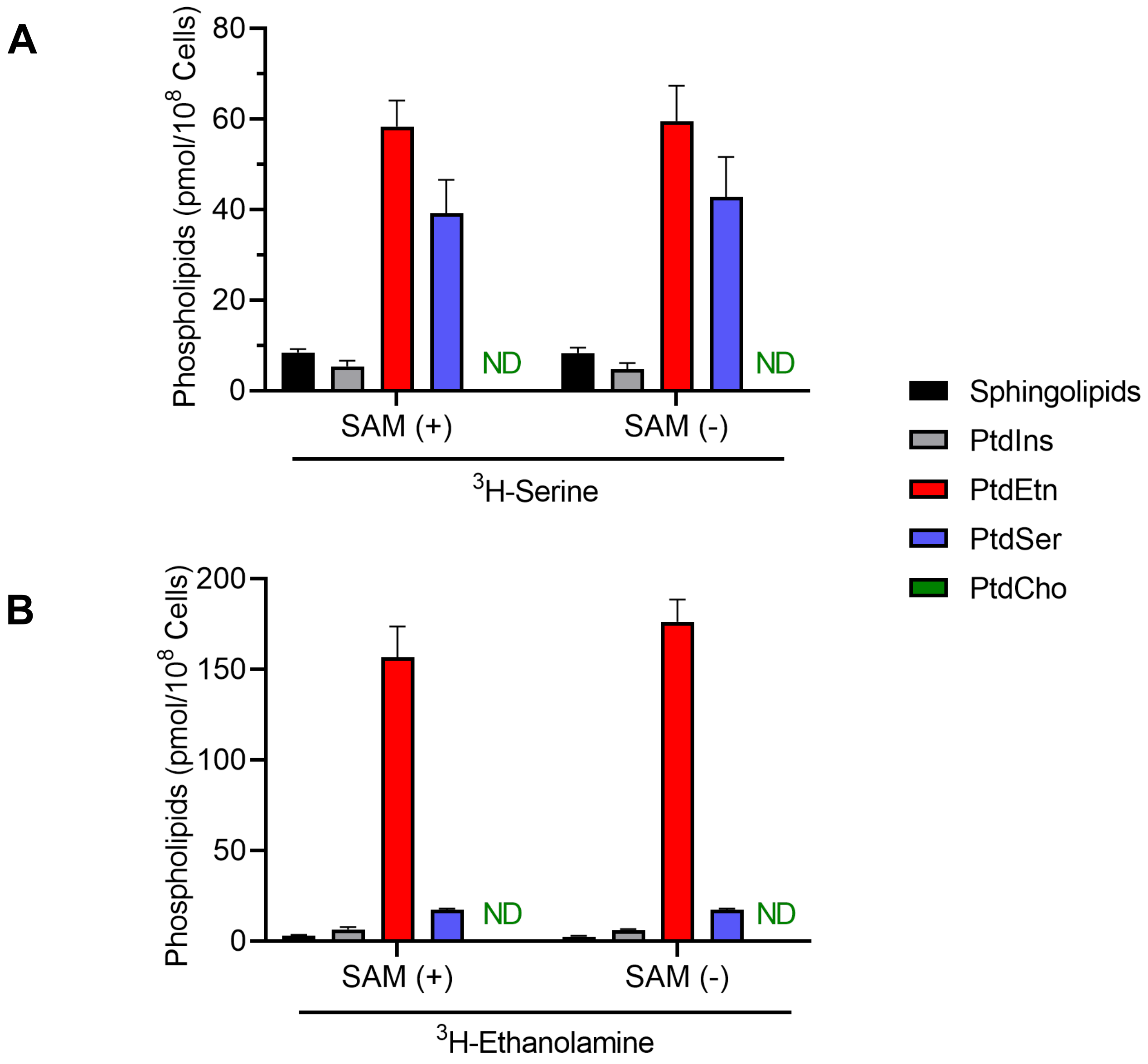

A
B
Phospholipids (pmol/10^8 Cells)
0
20
40
60
80
50
100
150
200
ND
SAM (+)
SAM (-)
3H-Serine
3H-Ethanolamine
Sphingolipids
PtdIns
PtdEtn
PtdSer
PtdCho

Figure S4

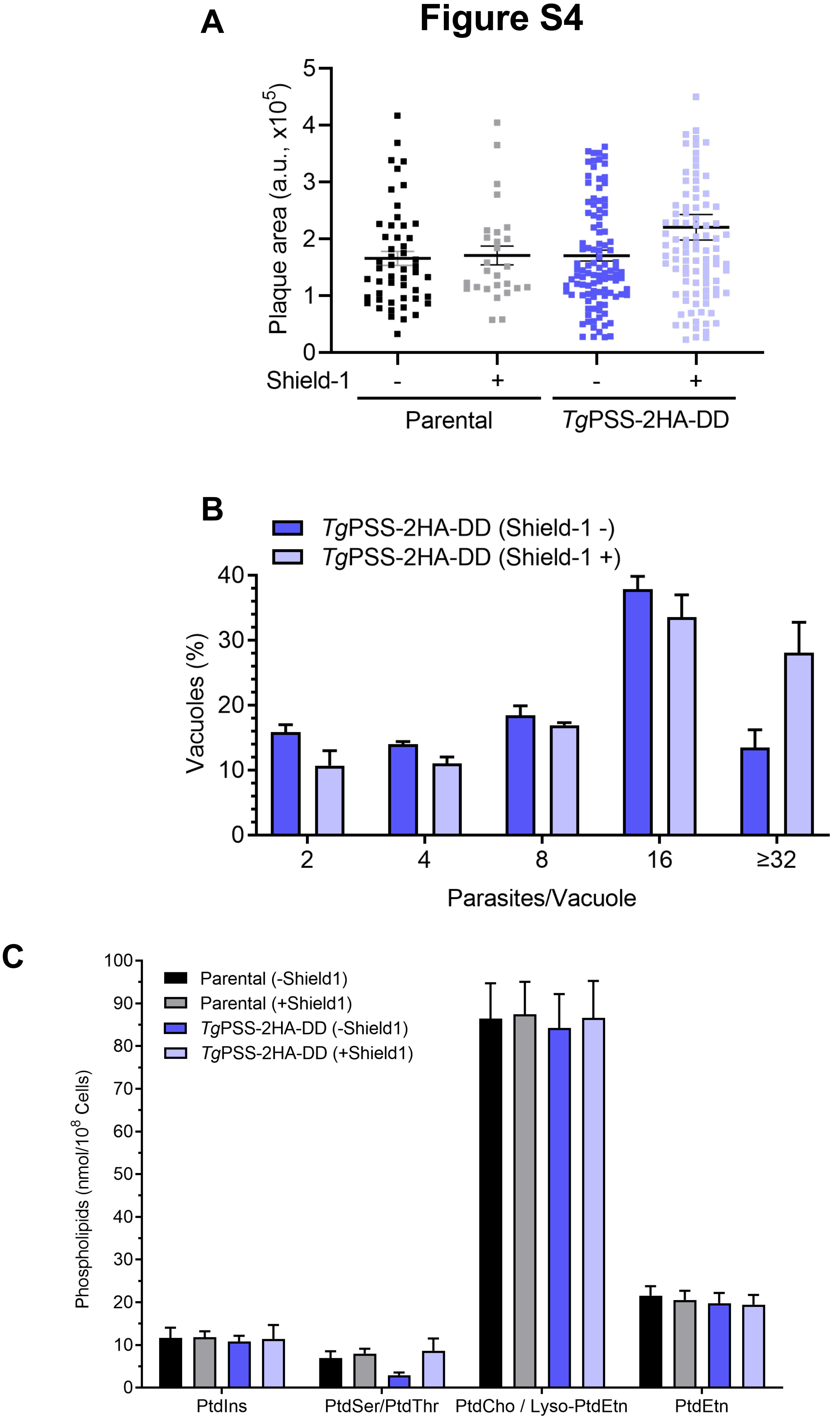
A
Plaque area (a.u., x10^5)
0
1
2
3
4
5
Shield-1
-
+
-
+
Parental
TgPSS-2HA-DD
B
TgPSS-2HA-DD (Shield-1 -)
TgPSS-2HA-DD (Shield-1 +)
Vacuoles (%)
0
10
20
30
40
2
4
8
16
≥32
Parasites/Vacuole
C
Phospholipids (nmol/10^8 Cells)
0
10
20
30
40
50
60
70
80
90
100
Parental (-Shield1)
Parental (+Shield1)
TgPSS-2HA-DD (-Shield1)
TgPSS-2HA-DD (+Shield1)
PtdIns
PtdSer/PtdThr
PtdCho / Lyso-PtdEtn
PtdEtn

Figure S5

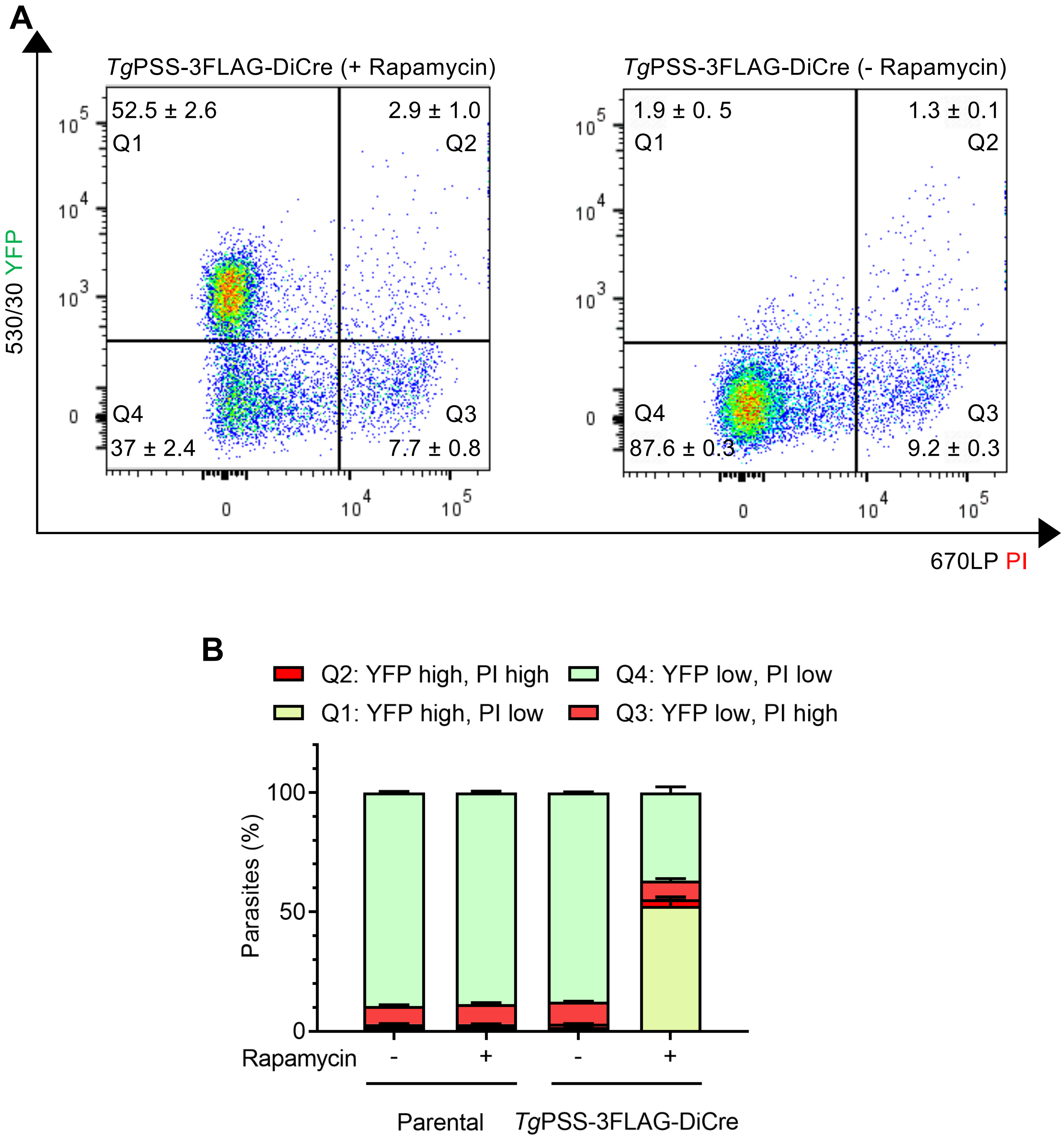
A
TgPSS-3FLAG-DiCre (+ Rapamycin)
TgPSS-3FLAG-DiCre (- Rapamycin)
52.5 ± 2.6
Q1
2.9 ± 1.0
Q2
Q4
37 ± 2.4
Q3
7.7 ± 0.8
1.9 ± 0. 5
Q1
1.3 ± 0.1
Q2
Q4
87.6 ± 0.3
Q3
9.2 ± 0.3
530/30 YFP
670LP PI
B
Q2: YFP high, PI high
Q4: YFP low, PI low
Q1: YFP high, PI low
Q3: YFP low, PI high
Parasites (%)
Rapamycin
Parental
TgPSS-3FLAG-DiCre

Figure S6

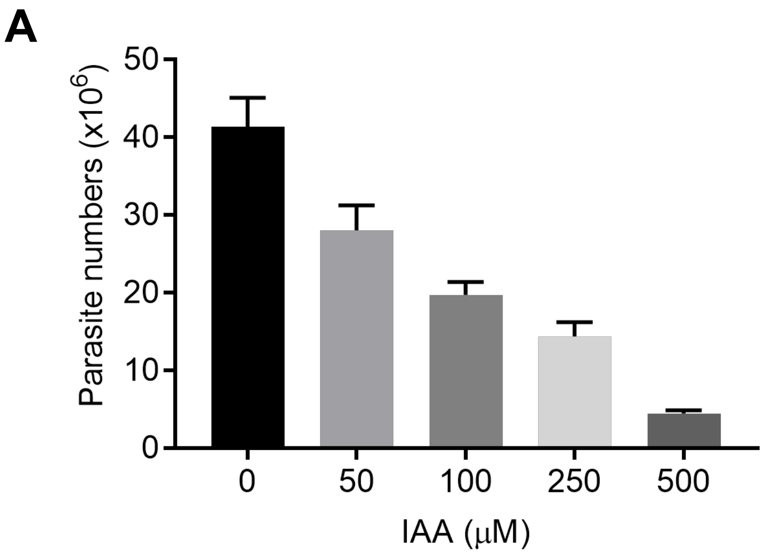

A
Parasite numbers (x10^6)
50
40
30
20
10
0
0
50
100
250
500
IAA (μM)

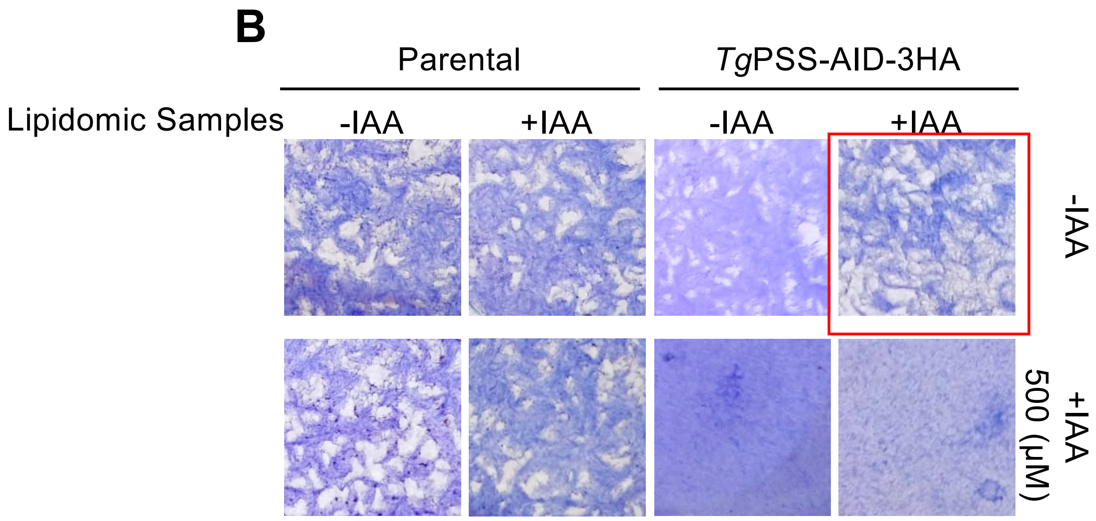

B
Parental
*Tg*PSS-AID-3HA
Lipidomic Samples
-IAA
+IAA
-IAA
+IAA
-IAA
+IAA
500 (μM)

Figure S7

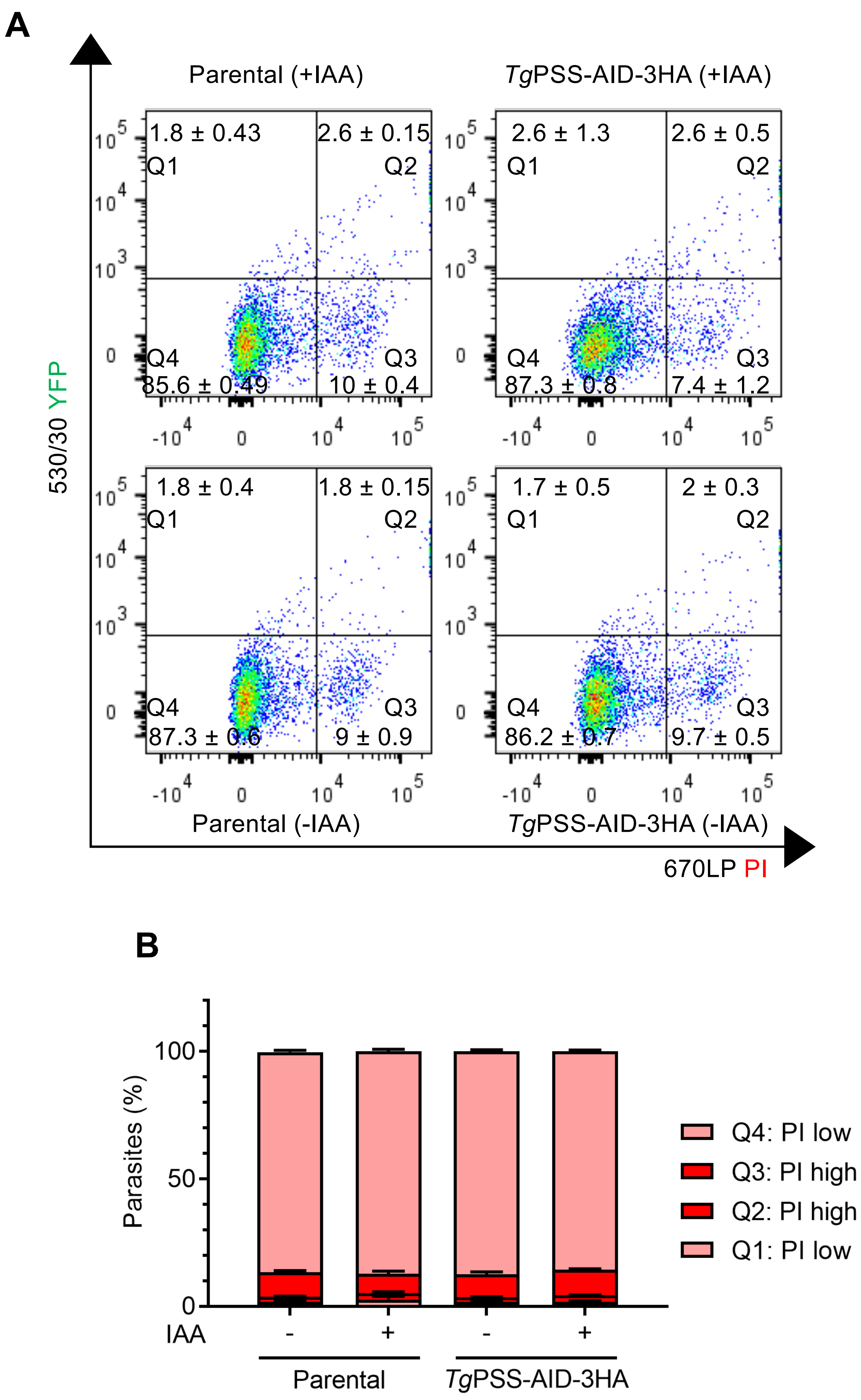
A
Parental (+IAA)
TgPSS-AID-3HA (+IAA)
1.8 ± 0.43
2.6 ± 0.15
85.6 ± 0.49
10 ± 0.4
2.6 ± 1.3
2.6 ± 0.5
87.3 ± 0.8
7.4 ± 1.2
1.8 ± 0.4
1.8 ± 0.15
87.3 ± 0.6
9 ± 0.9
1.7 ± 0.5
2 ± 0.3
86.2 ± 0.7
9.7 ± 0.5
Q1
Q2
Q3
Q4
Parental (-IAA)
TgPSS-AID-3HA (-IAA)
530/30 YFP
670LP PI
B
Parasites (%)
IAA
Parental
TgPSS-AID-3HA
Q4: PI low
Q3: PI high
Q2: PI high
Q1: PI low

Figure S8

A

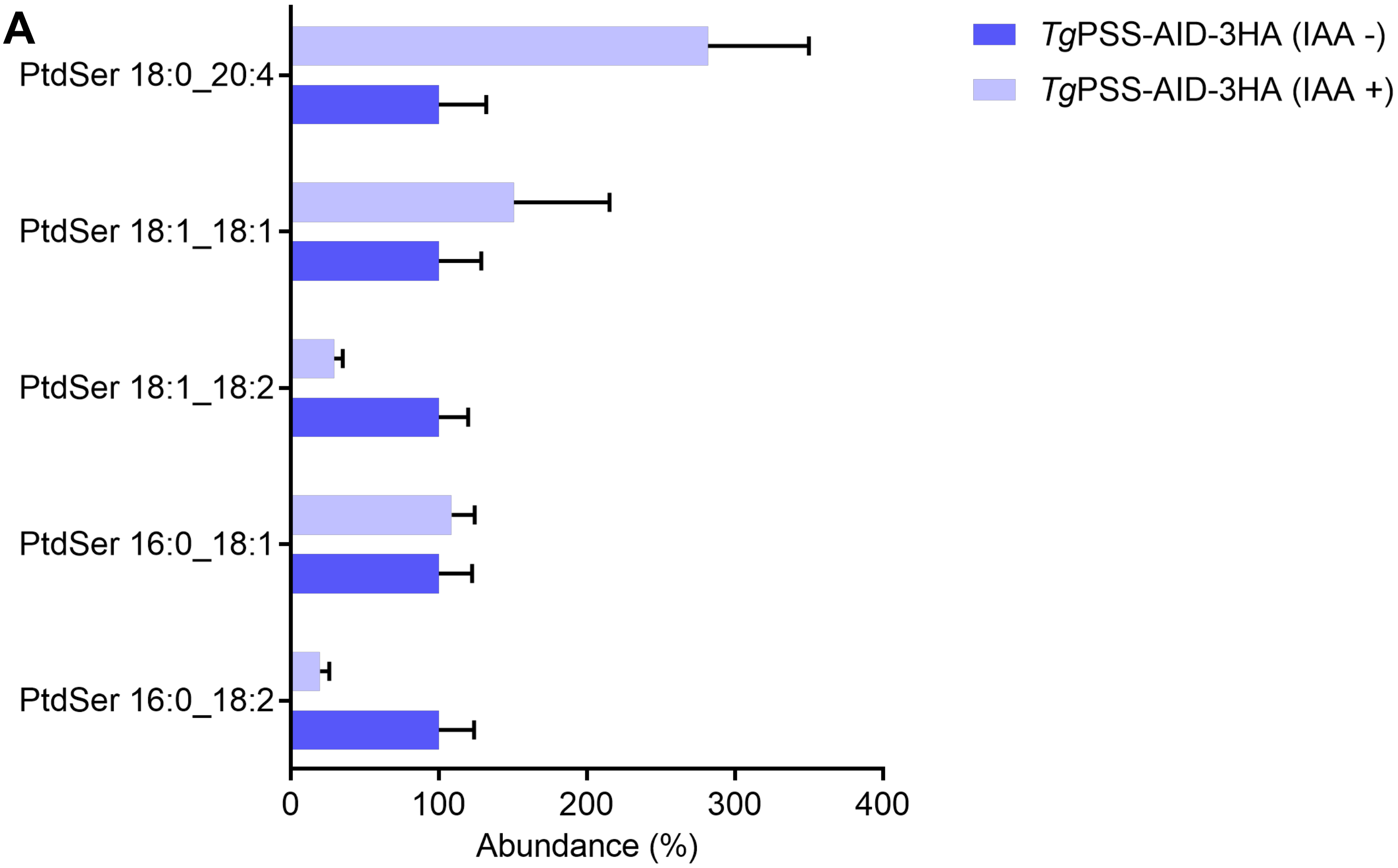
PtdSer 18:0_20:4
PtdSer 18:1_18:1
PtdSer 18:1_18:2
PtdSer 16:0_18:1
PtdSer 16:0_18:2
0
100
200
300
400
Abundance (%)
*Tg*PSS-AID-3HA (IAA -)
*Tg*PSS-AID-3HA (IAA +)

B

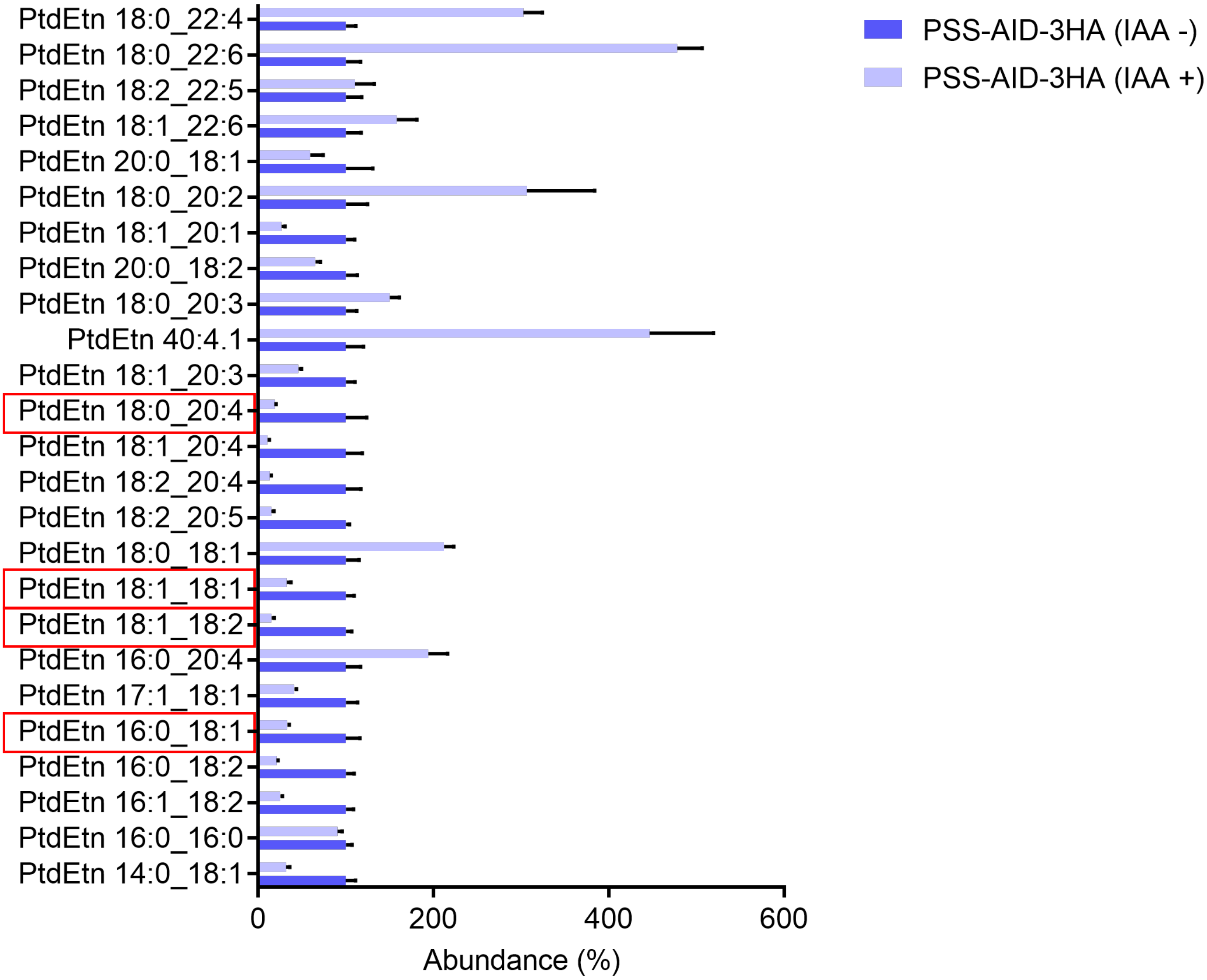
PtdEtn 18:0_22:4
PtdEtn 18:0_22:6
PtdEtn 18:2_22:5
PtdEtn 18:1_22:6
PtdEtn 20:0_18:1
PtdEtn 18:0_20:2
PtdEtn 18:1_20:1
PtdEtn 20:0_18:2
PtdEtn 18:0_20:3
PtdEtn 40:4.1
PtdEtn 18:1_20:3
PtdEtn 18:0_20:4
PtdEtn 18:1_20:4
PtdEtn 18:2_20:4
PtdEtn 18:2_20:5
PtdEtn 18:0_18:1
PtdEtn 18:1_18:1
PtdEtn 18:1_18:2
PtdEtn 16:0_20:4
PtdEtn 17:1_18:1
PtdEtn 16:0_18:1
PtdEtn 16:0_18:2
PtdEtn 16:1_18:2
PtdEtn 16:0_16:0
PtdEtn 14:0_18:1
0
200
400
600
Abundance (%)
PSS-AID-3HA (IAA -)
PSS-AID-3HA (IAA +)
